# TumorNext-Lynch-MMR: a comprehensive next generation sequencing assay for the detection of germline and somatic mutations in genes associated with mismatch repair deficiency and Lynch syndrome

**DOI:** 10.18632/oncotarget.24854

**Published:** 2018-04-17

**Authors:** Phillip N. Gray, Pei Tsai, Daniel Chen, Sitao Wu, Jayne Hoo, Wenbo Mu, Bing Li, Huy Vuong, Hsiao-Mei Lu, Navanjot Batth, Sara Willett, Lisa Uyeda, Swati Shah, Chia-Ling Gau, Monalyn Umali, Carin Espenschied, Mike Janicek, Sandra Brown, David Margileth, Lavinia Dobrea, Lawrence Wagman, Huma Rana, Michael J. Hall, Theodora Ross, Jonathan Terdiman, Carey Cullinane, Savita Ries, Ellen Totten, Aaron M. Elliott

**Affiliations:** ^1^ Advanced Genomic Services, Ambry Genetics, Aliso Viejo, CA 92656, USA; ^2^ Clinical Diagnostics Department, Ambry Genetics, Aliso Viejo, CA 92656, USA; ^3^ Bioinformatics Department, Ambry Genetics, Aliso Viejo, CA 92656, USA; ^4^ Cancer Genetic Risk Assessment Program, Arizona Oncology, Scottsdale, AZ 85258, USA; ^5^ Cancer Genetics Program, Saint Joseph of Orange, Orange, CA 92868, USA; ^6^ Oncology Research and Biospecimen Program, Saint Joseph of Orange, Orange, CA 92868, USA; ^7^ The Center for Cancer Prevention and Treatment, Saint Joseph of Orange, Orange, CA 92868, USA; ^8^ Department of Medical Oncology, Dana Farber Cancer Institute, Boston, MA 02461, USA; ^9^ Department of Clinical Genetics, Fox Chase Cancer Center, Philadelphia PA 19111, USA; ^10^ Department of Internal Medicine, University of Texas Southwestern Medical Center, Dallas, TX 75390, USA; ^11^ Department of Medicine – Gastroenterology, University of California San Francisco, San Francisco, CA 94115, USA; ^12^ Department of Pathology, Long Beach Memorial Medical Center, Long Beach, CA 90801, USA; ^13^ Advocate Medical Group, Park Ridge, Illinois 60068, USA

**Keywords:** Lynch syndrome, colorectal cancer, microsatellite instability, mismatch repair deficiency, next generation sequencing

## Abstract

The current algorithm for Lynch syndrome diagnosis is highly complex with multiple steps which can result in an extended time to diagnosis while depleting precious tumor specimens. Here we describe the analytical validation of a custom probe-based NGS tumor panel, TumorNext-Lynch-MMR, which generates a comprehensive genetic profile of both germline and somatic mutations that can accelerate and streamline the time to diagnosis and preserve specimen. TumorNext-Lynch-MMR can detect single nucleotide variants, small insertions and deletions in 39 genes that are frequently mutated in Lynch syndrome and colorectal cancer. Moreover, the panel provides microsatellite instability status and detects loss of heterozygosity in the five Lynch genes; *MSH2*, *MSH6*, *MLH1*, *PMS2* and *EPCAM*. Clinical cases are described that highlight the assays ability to differentiate between somatic and germline mutations, precisely classify variants and resolve discordant cases.

## INTRODUCTION

There are several types of hereditary colorectal cancer (CRC) syndromes, which account for 5-10% of all CRC cases [1]. The most common type, Lynch syndrome (also known as hereditary nonpolyposis colorectal cancer, or HNPCC), is characterized by germline mutations in the DNA mismatch repair (MMR) genes (*MLH1, MSH2, MSH6* and *PMS2*) or deletion of the 3’ end of *EPCAM*. Estimated cancer risks associated with germline mutations vary widely by gene and study and range from 10-83% for CRC and 16-62% for endometrial cancers [2–4]. Lynch syndrome has been estimated to occur in 1 in 279 individuals in the general population [5].

In the past, testing for Lynch syndrome was limited to patients that met either Amsterdam criteria or Bethesda guidelines; however, these guidelines have been shown to miss up to 89% and 63% of individuals with Lynch syndrome, respectively [6–9]. Due to the limitations of these criteria, the high prevalence of Lynch syndrome, and the availability of interventions to reduce cancer risk and mortality in mutation carriers, universal screening of all CRCs and endometrial cancers using immunohistochemistry (IHC) and/or microsatellite instability (MSI) testing has been implemented at many hospitals across the United States and is recommended by several professional societies [7–14].

**Figure 1:**
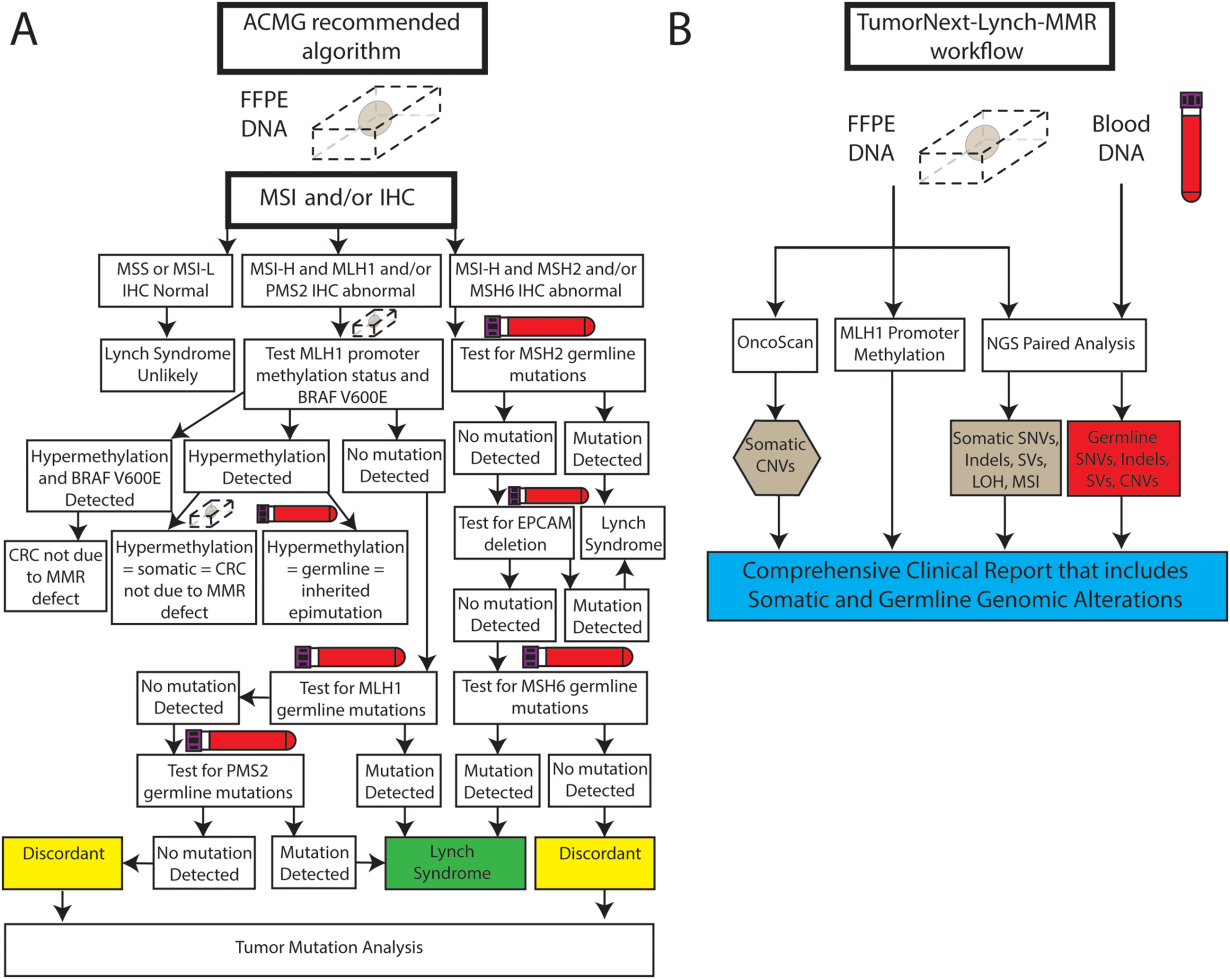
**(A)** Representation of the suggested ACMG algorithm for Lynch syndrome testing. Figure adopted from “ACMG technical standards and guidelines for genetic testing for inherited colorectal cancer (Lynch syndrome, familial adenomatous polyposis, and MYH-associated polyposis)”, Genetics in Medicine (2014) 16, 101-116 (https://doi.org/10.1038/gim.2013.166). **(B)** TumorNext-Lynch-MMR workflow. Paired analysis of blood and tumor is performed to detect germline and somatic SNVs, indels, SVs and somatic MSI and LOH. Tumor tissue is also analyzed for MLH1 promoter hypermethylation and CNVs.

Based on these guidelines, if IHC shows loss of MLH1 protein expression, testing for *BRAF* V600E (specific to colon cancer) or methylation of the *MLH1* promoter should be performed. If that is normal or IHC shows loss of the other MMR proteins, targeted germline gene testing is recommended. If germline MMR gene analysis identifies a mutation, a diagnosis of Lynch syndrome is made; however, due to the multiple steps and possibility of several testing outcomes, the algorithm is complex and can potentially become convoluted, time consuming and expensive (Figure 1A) [15]. The iterative nature of the algorithm also contributes to undue burden on patients, which may lead to testing fatigue and loss to follow-up. In addition, MSI and IHC may not detect 17-23% of patients with Lynch syndrome and in 52-59% of cases with abnormal MSI and/or IHC who proceed to germline analysis, a germline mutation may not be identified [16, 17]. These discordant results have complicated patient management for years, as the abnormal MSI/IHC results dictated that patients be managed as though they had Lynch syndrome, even in the absence of a germline mutation. More recent data, however, has shown that up to 70% of these cases may be explained by the presence of two acquired somatic mutations in one of the MMR genes [18]. The identification of two somatic MMR mutations that explain absent IHC results (i.e. lack of protein staining), in the absence of a germline mutation in that gene, can drastically change a patient’s management from that of an individual with presumed Lynch syndrome and high risk for second primary cancers to that of the average person with a personal history of cancer. This includes fewer colonoscopies, no need for consideration of risk-reducing surgery or screening for other Lynch syndrome associated cancers, and means that the patient’s and their family members’ cancer risks are based on their personal and family history.

Identifying MMR deficiency also has implications for therapy. Patients with stage II MMR deficient CRC have a good prognosis and do not benefit from fluorouracil-based chemotherapy [19]. In addition, recent advances in immunotherapy have provided another reason to identify patients with MMR deficiency due to both germline and somatic mutations. Patients with MMR deficient metastatic CRC were shown to respond to the PD-1 inhibitor pembrolizumab [20]. A later study showed response to pembrolizumab in patients with MMR deficient solid tumors of various types, leading to FDA approval of this drug for the treatment of MMR deficient metastatic solid tumors of any type [21]. Also, the FDA recently approved nivolumab, another PD-1 inhibitor, for MSI-H or MMR deficient metastatic CRC tumors [22].

Here we describe the validation of a custom probe-based next generation sequencing (NGS) panel that targets all exons and select intronic regions of the MMR genes, which facilitates accurate detection of single nucleotide variants (SNVs), copy neutral loss of heterozygosity (LOH), and exon-level resolution copy number variants (CNVs) from paired tumor and blood specimens. The panel also analyzes other genes recommended in the work up of metastatic CRC, such as *BRAF*, *KRAS* and *NRAS* [23]. This assay bypasses the standard Lynch syndrome diagnostic algorithm and provides a comprehensive analysis of somatic and germline MMR mutations, efficiently diagnosing or ruling out Lynch syndrome and providing guidance for therapy in one step (Figure 1B). Also described are several case studies that highlight the assay’s ability to resolve discordant cases, reclassify variants, and determine a final diagnosis.

## RESULTS

The assay consists of a hybridization-based capture/target enrichment and sequence analysis using massively parallel sequencing. CNVs were determined by the Affymetrix OncoScan and NGS [24]. DNA was extracted from both formalin-fixed-paraffin-embedded (FFPE) tissue and blood and dual analysis was performed to differentiate between germline and somatic mutations. Four datasets were generated to determine analytical sensitivity and specificity for the TumorNext-Lynch-MMR panel; SNVs/indels (somatic – 58 samples), SNVs/indels (germline – 5 samples), MSI (somatic – 104 samples) and LOH (somatic – 8 samples). The TumorNext-Lynch-MMR panel gene list is in Supplementary Table 1.

### Somatic and germline variant detection

A total of 209 variants in 58 samples were used to determine the analytical sensitivity and specificity in somatic variant detection. Genomic DNA was isolated from both FFPE tissue and peripheral blood from specimens previously characterized on TumorNext. Overall, mutation frequencies detected using TumorNext matched the frequencies obtained with TumorNext-Lynch-MMR (Supplementary Table 2). There were several variant calls in multiple samples that were not detected in either the original TumorNext run or TumorNext-Lynch-MMR run. These calls were not detected by the bioinformatics pipeline due to low coverage (below 100x), low frequency (below 5%), or both in the tumor sample. The majority of these discordant calls were resolved by reviewing sequence alignment data using the Integrative Genomics Viewer [25]; however, 3 calls were not resolved due to insufficient coverage or presumed tumor heterogeneity. Specimen RD_W13_1352G11 was discordant for the mutation *SMAD4* S232Qfs^*^3, which was detected at 5.45% by Tumor-Lynch-MMR at coverage 374x and absent from the TumorNext data. This region was only covered at 49x in the TumorNext data, which is not enough coverage to detect variants at ∼5%. Specimen BR_15_23_05_01_T was discordant for the mutation *MLH1* c.1039-5T>G, which was detected at 5.85% on TumorNext and absent from the TumorNext-Lynch-MMR data. This region was only covered at 98x, which is not enough coverage to detect variants at ∼5%. Specimen BR_14_209_05_03_T was discordant for the mutation *KIT* Y721C, which was detected at 11.9% on TumorNext and absent from the TumorNext-Lynch-MMR data. This region was not covered by any reads on the TumorNext-Lynch-MMR capture, so it was not included in the sensitivity calculation.

In total, 274 genotypes were determined with 2 false negatives and 1 potential false positive. Based on these results, the analytical sensitivity (True Positives/(True Positives + False Negatives) of the TumorNext-Lynch-MMR panel was 252/252 + 2 = 99.21% (95% CI, 96.9%-99.9%) and the analytical specificity (True Negatives/True Negatives + False Positives) of TumorNext-Lynch-MMR for the 477,945bp exon panel (or 27,720,810bp for 58 samples) is [27,720,810/(27,720,810 + 1)] = 99.99% (95% CI, 99.99%-100%). The potential false positive in Specimen RD_W13_1352G11 data is likely a true call as the region did not have enough sequencing coverage in the original TumorNext data to detect the variant, which is at the lower limit of detection (∼5%).

To measure accuracy for calling germline variants, 5 clinical samples containing 80 SNPs from consenting patients previously analyzed on CancerNext were tested with the TumorNext-Lynch-MMR assay and results compared [26]. All variants detected by CancerNext were also detected by TumorNext-Lynch-MMR (Supplementary Table 3). Based on these results, the analytical sensitivity is [80/(80 + 0)] = 100% (95% CI, 94.3%-100%) with a false negative rate of 0% and analytical specificity for the 229,050bp region analyzed (5 samples x 45,810bp region = 229,050bp)is [229,050/(229,050 + 0)] = 100% (95% CI, 99.99%-100%) with a false positive rate of 0%.

### Microsatellite instability detection

Microsatellite instability is a phenomenon observed in tumors cells when genes involved in MMR are mutated. MSI is detected by analyzing homopolymer regions for insertions or deletions in DNA extracted from tumor cells and compared to DNA extracted from normal cells [27–29]. The Bethesda guidelines outlined a classification scheme based on five microsatellite markers where samples are designated MSI-High (MSI-H) if two or more markers are unstable, MSI-Low (MSI-L) if one marker is unstable and MSI-Stable (MSS) if all markers are unchanged [30]. Kim *et al.* examined 277 CRC and endometrial cancer (EC) samples from the TCGA dataset for MSI and determined the number of events between MSI-L and MSS tumors was not significant; MSI-L tumors had an average of 5 and 2 events, and stable tumors had 4 and 1 events for CRC and EC, respectively. Endometrial cancer tumors also exhibited fewer MSI events than CRC (126 vs 290) overall [31]. Moreover, EC tumors exhibit more subtle changes in homopolymer lengths compared to CRC tumors [32]. These observations suggest detection of MSI in EC may be more challenging than CRC and minimal difference exists between MSI-L and stable tumors.

The TumorNext-Lynch-MMR panel contains 40 homopolymer regions that may be used for MSI detection using MSIsensor (Supplementary Table 4), a software designed specifically to detect MSI with NGS data [33]. Analysis of 104 tumor specimens previously characterized on the Promega MSI Analysis System (catalogue # MD1641) revealed a discordant rate of 2.88% with 2 false negatives and 1 false positive. Longer homopolymer regions were demonstrated to increase sensitivity of MSI detection [34]. In fact, the introduction of BAT-40 (which analyzes a mononucleotide region of 40 nucleotides) was shown to reclassify stable and MSI-L samples to MSI-L and MSI-H, respectively [35]. This may be due to an increased likelihood of DNA polymerase to introduce deletions from strand slippage with longer stretches of mononucleotides [36–39]. After review of the 40 homopolymer regions analyzed, it was determined their length only ranged from 12-20 nucleotides.

In an effort to increase sensitivity, we included the 5 “gold standard” MSI markers (NR-21, BAT-26, BAT-25, NR-24 and MONO-27) found in the Promega MSI Analysis System (catalogue # MD1641), which range 21-27 nucleotides, into the TumorNext-Lynch-MMR panel and tested the same samples on the updated panel. The discordant rate dropped to 1.92% and resolved the 1 false positive, but the 2 false negatives still remained. Overall, concordance was high (Table 1) with an analytical sensitivity (True Positives/(True Positives + False Negatives) of 51/51 + 2 = 96.22% (95% CI, 85.9%-99.3%) and the analytical specificity (True Negatives/True Negatives + False Positives) of [53 /(53 + 0)] = 100% (95% CI, 91.6%-100%). It is possible the 2 false negatives are the result of tumor heterogeneity as multiple DNA isolations were performed for these specimens.

**Table 1:** Concordance between NGS MSIsensor and Promega MSI Analysis

Sample ID	MSI (40 sites)	MSI (45 sites)	Promega Result
17_001	High	High	High
17_002	High	High	High
17_003	High	High	High
17_004	High	High	High
17_005	High	High	High
17_006	High	High	High
17_007	High	High	High
17_008	High	High	High
17_009	High	High	High
17_010	High	High	High
17_011	Stable	Stable	High
17_012	High	High	High
17_013	High	High	High
17_014	High	High	High
17_015	High	High	High
17_016	High	High	High
17_017	High	High	High
17_018	High	High	High
17_019	High	High	High
17_020	High	High	High
17_021	High	High	High
17_022	Stable	Stable	Stable
17_023	Stable	Stable	Stable
17_024	Stable	Stable	Stable
17_025	Stable	Stable	Stable
17_026	Stable	Stable	Stable
17_027	Stable	Stable	Stable
17_028	Stable	Stable	Stable
17_029	Stable	Stable	Stable
17_030	Stable	Stable	Stable
17_031	Stable	Stable	Stable
17_032	High	High	High
17_033	Stable	Stable	Stable
17_034	High	High	High
17_035	High	High	High
17_036	High	High	High
17_037	High	High	High
17_038	High	Stable	Stable
17_039	High	High	High
17_040	High	High	High
17_041	High	High	High
17_042	High	High	High
17_043	High	High	High
17_044	Stable	Stable	Stable
17_045	Stable	Stable	Stable
17_046	High	High	High
17_047	High	High	High
17_048	High	High	High
17_049	High	High	High
17_050	Stable	Stable	Stable
17_051	High	High	High
17_052	High	High	High
17_053	Stable	Stable	Stable
BR14-43 05-01T	Stable	Stable	Stable
BR14-48 05-01T	Stable	Stable	Stable
BR14-13 05-01T	Stable	Stable	Stable
BR14-131 05-01T	Stable	Stable	Stable
BR14-239 05-01T	Stable	Stable	Stable
BR13-102T	Stable	Stable	Stable
BR-14-248_05-02	Stable	Stable	Stable
BR-14-48_05-02T	Stable	Stable	Stable
BR-15-183_05-1	Stable	Stable	Stable
BR-15-57_05-01	Stable	Stable	Stable
BR-14-283_05-01	Stable	Stable	Stable
BR11-49 396-4T	Stable	Stable	Stable
BR11-93 T	Stable	Stable	Stable
BR12-11 1041-3T	Stable	Stable	Stable
BR12-15 T	Stable	Stable	Stable
BR13-162 05-01T	Stable	Stable	Stable
BR13-163 T	Stable	Stable	Stable
BR13-191 05-01T	Stable	Stable	Stable
BR13-184 05-01T	Stable	Stable	Stable
BR13-25 05-01T	Stable	Stable	Stable
BR11-71 T	Stable	Stable	Stable
NP-17963 T	High	High	High
NP-18023 T	High	High	High
NP-18212 T	High	High	High
NP-18215 T	High	High	High
BR14-209 05-01T	High	High	High
BR13-29 05-01T	High	High	High
BR-14-267_05-02	Stable	Stable	High
BR-14-202_05-1	High	High	High
BR-13-170_05-01T	High	High	High
BR-15-12_05-01	High	High	High
BR-14-312_05-01	High	High	High
BR-15-37_05-01	High	High	High
BR12-110 T	High	High	High
BR12-30 T	High	High	High
BR14-88 05-01T	Stable	Stable	Stable
BR14-194 05-01T	Stable	Stable	Stable
BR-14-51_05-01T	Stable	Stable	Stable
BR-15-168_05-01	Stable	Stable	Stable
BR-14-138_05-02T	Stable	Stable	Stable
BR-15-90_05-01	Stable	Stable	Stable
BR-14-253_05-02	Stable	Stable	Stable
BR-14-97_07-01N	Stable	Stable	Stable
BR-13-187_05-02T	Stable	Stable	Stable
BR-14-20_05-01T	Stable	Stable	Stable
BR-14-231_05-03T	Stable	Stable	Stable
BR-14-257_05-03	Stable	Stable	Stable
BR-14-293_05-01	Stable	Stable	Stable
BR-15-23_05-01	Stable	Stable	Stable
BR-15-70 _05-01	Stable	Stable	Stable
BR-12-37 1265_3	Stable	Stable	Stable
**Disconcordant**	3	2	
**Total**	104	104	
**Disconcordant rate**	2.88%	1.92%	
			True positives = 51
			True negatives = 53
For the Promega kit, status was determined to be MSI-High if 2 or more markers were expanded and Stable if one or fewer markers were expanded.			
Criteria	MSI (40 sites)	MSI (45 sites)	
Failed	<35 sites with coverage	<35 sites with coverage	
Stable	< 20% Expanded	< 20% Expanded	
High	≥ 20% Expanded	≥ 20% Expanded	

### Loss of heterozygosity detection

Loss of heterozygosity in tumors with mutations in MLH1, MSH2, MSH6 and PMS2 will lead to the inactivation of both alleles if the wild type copy is lost [40]. This “second hit” occurs at a high frequency, so the TumorNext-Lynch-MMR panel was designed to detect LOH by incorporating intronic regions of *MSH2*, *MSH6*, *MLH1*, *PMS2* and *EPCAM* and measuring allelic imbalances [41, 42]. We identified specimens in our tumor repository that exhibited LOH in one of the 5 Lynch syndrome genes based on analysis using the Affymetrix OncoScan. These were used to determine sensitivity and specificity of LOH detection using intronic SNP allele frequencies.

Overall, accuracy was high when comparing OncoScan to NGS data to determine LOH (Table 2) with few discrepancies. Sample BR14_26, was reported to have LOH in *PMS2* by OncoScan that was not detected by NGS, however, only ∼20% of cells are expected to have LOH. As a result, heterozygous allele frequencies are predominant in this region which will not result in detection of LOH by the NGS pipeline. This sample is highly heterogeneous with more than one dominant genotype. A second sample, BR15_168, which was used in both intra- and inter- validation runs produced one false positive in *PMS2* for 1 of the 5 runs. The % tumor for this sample is 35% and the copy number for the region is 3, so some allelic imbalance is expected. Based on this analysis, it appears the NGS pipeline requires >20% of the tumor DNA to contain the region of LOH for the pipeline to accurately detect it. The one false positive call resulted from a region with a copy number of 3 in approximately 20% of the tumor DNA.

**Table 2:** Concordance between NGS and OncoScan for LOH detection

NGS Sample ID	Gene	OncoScan CN/LOH (+/-)	NGS CN/LOH (+/-)	Notes
BR_14_231_05_03T_Val2	PMS2	5/-	3/-	
	EPCAM	3/-	2/-	
	MLH1	3/-	2/-	
	MSH2	3/-	2/-	
	MSH6	3/-	2/-	
BR_14_248_05_02T_Val2	PMS2	4/+ 100% LOH	2/+ LOH	
	EPCAM	2/-	2/-	
	MLH1	3/-	2/-	
	MSH2	2/-	2/-	
	MSH6	4/+ 100% LOH	2/+ LOH	
BR_14_248_05_02T_Val3	PMS2	4/+ 100% LOH	2/+ LOH	
	EPCAM	2/-	2/+ LOH	
	MLH1	3/-	2/-	
	MSH2	2/-	2/+ LOH	
	MSH6	4/+ 100% LOH	2/+ LOH	
BR_15_168_05_01_T_INTRA1_Val1	PMS2	3/-	2/+ LOH	False Positive/Allelelic imbalance due to CN = 3
	EPCAM	2/+ 50% LOH	2/+ LOH	50% of tumor cells contain LOH
	MLH1	2/-	2/-	
	MSH2	2/+ 50% LOH	2/+ LOH	50% of tumor cells contain LOH
	MSH6	2/+ 50% LOH	2/+ LOH	50% of tumor cells contain LOH
BR_15_168_05_01_T_INTRA2_Val1	PMS2	3/-	2/-	
	EPCAM	2/+ 50% LOH	2/+ LOH	50% of tumor cells contain LOH
	MLH1	2/-	2/-	
	MSH2	2/+ 50% LOH	2/+ LOH	50% of tumor cells contain LOH
	MSH6	2/+ 50% LOH	2/+ LOH	50% of tumor cells contain LOH
BR_15_168_05_01_T_INTRA3_Val1	PMS2	3/-	2/-	
	EPCAM	2/+ 50% LOH	2/+ LOH	50% of tumor cells contain LOH
	MLH1	2/-	2/-	
	MSH2	2/+ 50% LOH	2/+ LOH	50% of tumor cells contain LOH
	MSH6	2/+ 50% LOH	2/+ LOH	50% of tumor cells contain LOH
BR_15_168_05_01_T_Val2	PMS2	3/-	2/-	
	EPCAM	2/+ 50% LOH	2/+ LOH	50% of tumor cells contain LOH
	MLH1	2/-	2/-	
	MSH2	2/+ 50% LOH	2/+ LOH	50% of tumor cells contain LOH
	MSH6	2/+ 50% LOH	2/+ LOH	50% of tumor cells contain LOH
BR_15_90_05_01_T_Val1	PMS2	4/-	2/-	
	EPCAM	3/-	2/-	
	MLH1	3/+ 50% LOH	2/+ LOH	50% of tumor cells contain LOH
	MSH2	3/-	2/-	
	MSH6	3/-	2/-	
BR_15_90_05_01_T_Val2	PMS2	4/-	2/-	
	EPCAM	3/-	2/-	
	MLH1	3/+ 50% LOH	2/+ LOH	50% of tumor cells contain LOH
	MSH2	3/-	2/-	
	MSH6	3/-	2/-	
BR_15_90_05_01_T_Val3	PMS2	4/-	2/-	
	EPCAM	3/-	2/-	
	MLH1	3/+ 50% LOH	2/+ LOH	50% of tumor cells contain LOH
	MSH2	3/-	2/-	
	MSH6	3/-	2/-	
BR11_71_575_3_T_Val1	PMS2	2/-	2/-	
	EPCAM	2/-	2/-	
	MLH1	2/-	2/-	
	MSH2	2/-	2/-	
	MSH6	2/-	2/-	
BR11_71_575_3_T_Val2	PMS2	2/-	2/-	
	EPCAM	2/-	2/-	
	MLH1	2/-	2/-	
	MSH2	2/-	2/-	
	MSH6	2/-	2/-	
BR11_71_575_3_T_Val3	PMS2	2/-	2/-	
	EPCAM	2/-	2/-	
	MLH1	2/-	2/-	
	MSH2	2/-	2/-	
	MSH6	2/-	2/-	
BR13_102_05_01T_Val1	PMS2	2/-	2/-	
	EPCAM	2/-	2/-	
	MLH1	2/-	2/-	
	MSH2	2/-	2/-	
	MSH6	2/-	2/-	
BR13_102_05_01T_Val2	PMS2	2/-	2/-	
	EPCAM	2/-	2/-	
	MLH1	2/-	2/-	
	MSH2	2/-	2/-	
	MSH6	2/-	2/-	
BR13_102_05_01T_Val3	PMS2	2/-	2/-	
	EPCAM	2/-	2/-	
	MLH1	2/-	2/-	
	MSH2	2/-	2/-	
	MSH6	2/-	2/-	
BR13_116_05_01T_Val1	PMS2	2/-	2/-	
	EPCAM	2/-	2/-	
	MLH1	1/+ 30% LOH	1/+ LOH	30% of tumor cells contain LOH
	MSH2	2/-	2/-	
	MSH6	2/-	2/-	
BR13_116_05_01T_Val2	PMS2	2/-	2/-	
	EPCAM	2/-	2/-	
	MLH1	1/+ 30% LOH	1/+ LOH	30% of tumor cells contain LOH
	MSH2	2/-	2/-	
	MSH6	2/-	2/-	
BR13_116_05_01T_Val3	PMS2	2/-	2/-	
	EPCAM	2/-	2/-	
	MLH1	1/+ 30% LOH	1/+ LOH	30% of tumor cells contain LOH
	MSH2	2/-	2/-	
	MSH6	2/-	2/-	
BR13_191_05_01_T_Val1	PMS2	3/-	3/-	
	EPCAM	2/-	2/-	
	MLH1	2/-	2/-	
	MSH2	2/-	2/-	
	MSH6	2/-	2/-	
BR13_191_05_01_T_Val2	PMS2	3/-	3/-	
	EPCAM	2/-	2/-	
	MLH1	2/-	2/-	
	MSH2	2/-	2/-	
	MSH6	2/-	2/-	
BR13_191_05_01_T_Val3	PMS2	3/-	3/-	
	EPCAM	2/-	2/-	
	MLH1	2/-	2/-	
	MSH2	2/-	2/-	
	MSH6	2/-	2/-	
BR13_81_05_01T_Val1	PMS2	2/-	2/-	
	EPCAM	2/-	2/-	
	MLH1	1/+ 30% LOH	1/+ LOH	30% of tumor cells contain LOH
	MSH2	2/-	2/-	
	MSH6	2/-	2/-	
BR13_81_05_01T_Val2	PMS2	2/-	2/-	
	EPCAM	2/-	2/-	
	MLH1	1/+ 30% LOH	1/+ LOH	30% of tumor cells contain LOH
	MSH2	2/-	2/-	
	MSH6	2/-	2/-	
BR13_81_05_01T_Val3	PMS2	2/-	2/-	
	EPCAM	2/-	2/-	
	MLH1	1/+ 30% LOH	1/+ LOH	30% of tumor cells contain LOH
	MSH2	2/-	2/-	
	MSH6	2/-	2/-	
BR13_97_T_Val1	PMS2	3/-	4/-	
	EPCAM	2/-	3/-	
	MLH1	1/+ 100% LOH	1/+ LOH	
	MSH2	2/-	3/-	
	MSH6	2/-	3.5/-	
BR13_97_T_Val2	PMS2	3/-	4/-	
	EPCAM	2/-	3/-	
	MLH1	1/+ 100% LOH	1/+ LOH	
	MSH2	2/-	3/-	
	MSH6	2/-	3.5/-	
BR13_97_T_Val3	PMS2	3/-	4/-	
	EPCAM	2/-	3/-	
	MLH1	1/+ 100% LOH	1/+ LOH	
	MSH2	2/-	3/-	
	MSH6	2/-	3.5/-	
BR14_26_T_Val1	PMS2	2/+ 20% LOH	2/+ LOH	heterozygous allele frequencies are predominant in this region which will not result in detection of LOH by the NGS pipeline
	EPCAM	2/-	2/-	
	MLH1	2/-	2/-	
	MSH2	2/-	2/-	
	MSH6	2/-	2/-	
BR14_26_T_Val2	PMS2	2/+ 20% LOH	2/+ LOH	heterozygous allele frequencies are predominant in this region which will not result in detection of LOH by the NGS pipeline
	EPCAM	2/-	2/-	
	MLH1	2/-	2/-	
	MSH2	2/-	2/-	
	MSH6	2/-	2/-	
BR14_26_T_Val3	PMS2	2/+ 20% LOH	2/+ LOH	heterozygous allele frequencies are predominant in this region which will not result in detection of LOH by the NGS pipeline
	EPCAM	2/-	2/-	
	MLH1	2/-	2/-	
	MSH2	2/-	2/-	
	MSH6	2/-	2/-	

Note: %LOH was determined by reviewing BAF plots in Nexus. Heterozygous samples display data points aggregated at 1, 0.5 and 0, forming 3 distinct data “bands”. LOH samples exhibit 2 distinct data bands with aggregated data points at 1 and 0. %LOH is less than 100% as data points form 4 distinct bands, separating from the 0 and 1 positions. The closer the band is to 0.5, the less % LOH (and closer to the heterozygous state of 3 data bands). It appears that >20% of the sample must exhibit LOH for the NGS pipeline to detect LOH. Based on this observation, the limit of LOH detection was set at 30%.

In total, 155 regions (5 genes analyzed from 31 independent sequenced samples = 155 total regions) were analyzed for LOH. Based on these results, the analytical sensitivity (True Positives/(True Positives + False Negatives) of the TumorNext-Lynch-MMR panel was 32/32 + 3 = 91.4% (95% CI, 75.8%-97.7%) and the analytical specificity (True Negatives/True Negatives + False Positives) of TumorNext-Lynch-MMR for the is [123 /(123 + 1)] = 99.2% (95% CI, 94.9%-99.9%).

The 3 false negatives were from the same sample that was sequenced three times. The sample, BR14_26, was reported to have LOH in *PMS2* by OncoScan that was not detected by NGS, however, only ∼20% of the tumor DNA is expected to have LOH. As a result, heterozygous allele frequencies are predominant in this region which will not result in detection of LOH by the NGS pipeline. This sample is highly heterogeneous with more than one dominant genotype. If this sample is excluded from the calculation, the analytical sensitivity is [152 /(152 + 0)] = 100%. Based on this observation, the limit of detection for LOH in tumor specimens is 30%.

### Precision and reproducibility

Previously characterized samples were used to assess intra- and inter-reproducibility for variant detection (germline and somatic), MSI and LOH. Samples were assayed in triplicate as intra- (1 sample) or inter-run replicates (10 samples) and were prepared separately by different technicians on multiple dates using non-redundant barcodes to minimize potential barcode bias. Overall, the expected germline and somatic variants, MSI status and LOH states were detected in each run. Moreover, somatic and germline variants were at similar frequencies between replicates (Supplementary Tables 5 - 12).

As mentioned previously, sample BR15_168 was used in both LOH intra- and inter- validation runs and produced one false positive in 1 of the 5 runs in the gene *PMS2* (Note: the sample failed QC in validation run 3, so the sample was omitted from the inter-assay dataset). This sample was at the lower limit of detection for the LOH pipeline as the percent tumor was 35% and approximately 20% of the tumor DNA was expected to have allelic imbalance. The intra-assay replicates also had reduced percent bases over 500x; the average percent bases over 500x is 74% for all tumor samples in the run (or 83% if 3 outliers are removed), but only 46%, 51% and 46% for intra-assay replicates 1-3, respectively. Moreover, LOH detection in *PMS2* may be complicated due to pseudogene issues.

## DISCUSSION

**Figure 2:**
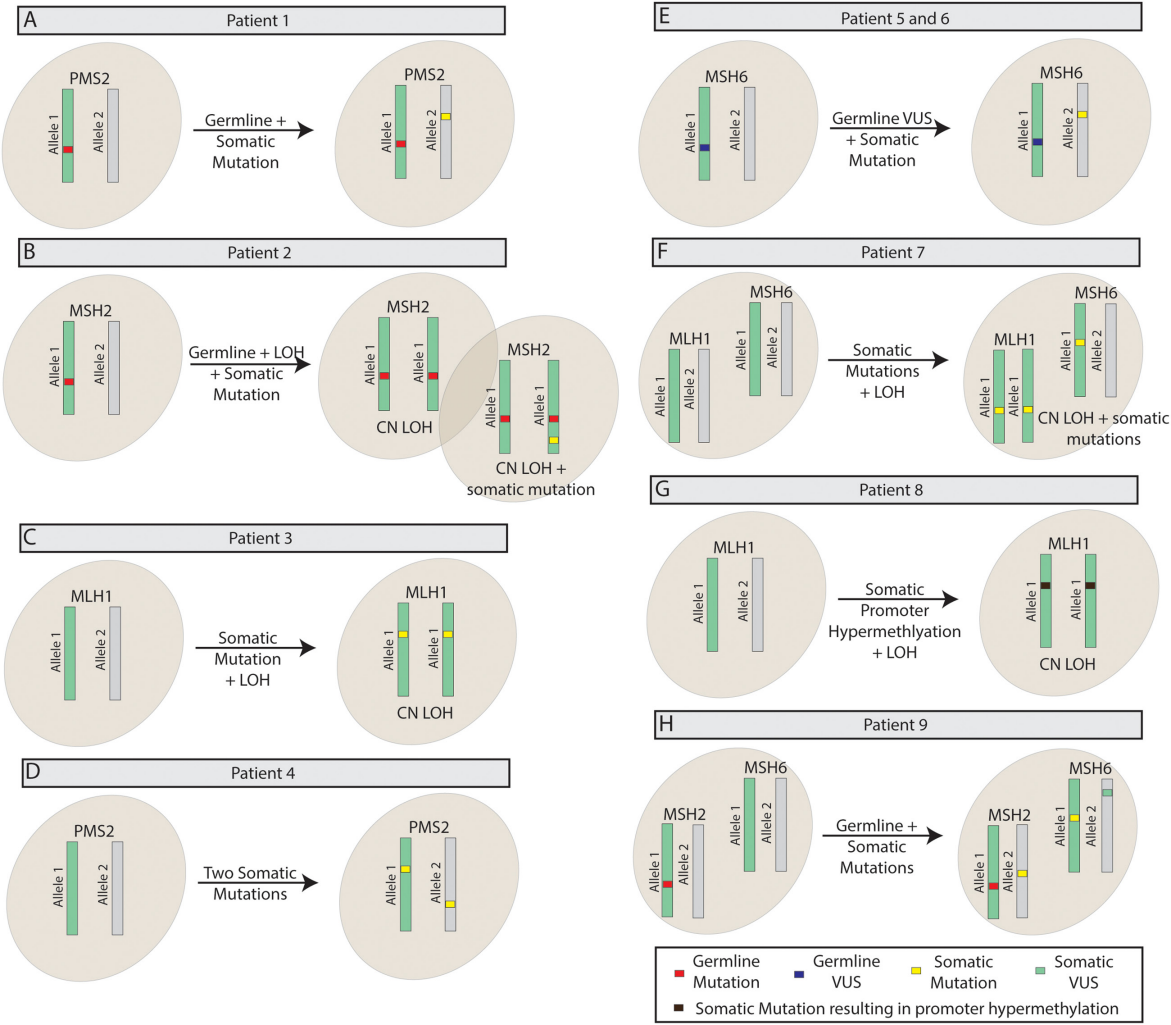
Representative patient scenarios observed since the launch of TumorNext-Lynch-MMR **(A)** Germline mutation + somatic mutation as the second hit, **(B)** Germline mutation + LOH as second hit, plus somatic mutation, **(C)** Somatic mutation + LOH, **(D)** Double somatic mutations, **(E)** Germline VUS + somatic mutation, **(F)** Multiple somatic mutations + LOH, **(G)** Somatic mutation leading to promoter hypermethylation + LOH, **(H)** Germline mutation + multiple somatic mutations.

TumorNext-Lynch-MMR was recently launched and most specimens received were from patients with clinical suspicion for Lynch syndrome. In the highlighted cases below, all were brought through the traditional Lynch syndrome testing algorithm only to require tumor sequencing to make a final diagnosis. If TumorNext-Lynch-MMR had been utilized upon initial diagnosis, significant time and costs may have been saved by stratifying patient risks and significantly reducing unnecessary cancer surveillance. Common scenarios with suspected Lynch syndrome cases are the absence of IHC staining for one or more MMR proteins and/or MSI-H without molecular evidence to make a Lynch syndrome diagnosis. A diagnosis of Lynch Syndrome can be made with the identification of a germline mutation. Tumor sequencing may reveal this mutation coupled to a somatic mutation in the opposite allele or in a state of LOH; either event would serve as the second hit. The former scenario is illustrated by patient 1, which had a history of endometroid adenocarcinoma of the uterus. IHC did not detect nuclear staining for PMS2 (i.e. absent protein expression in the nucleus), but did detect nuclear staining for MLH1, MSH2 and MSH6 (i.e. presence of proteins in the nucleus). TumorNext-Lynch-MMR revealed MSI-H and a pathogenic germline mutation (EX6_9del) and pathogenic somatic mutation (p.Y268^*^) in *PMS2* (Figure 2A). The later scenario is illustrated by patient 2, a 62 year old male with adenocarcinoma of the rectum diagnosed at 55 with a recurrent tumor. Patient had a family history of pancreatic cancer with mother and sister diagnosed at ages 53 and 51, respectively. IHC revealed absence of MSH2 and MSH6 protein and presence of PMS2 and MLH1 protein. TumorNext-Lynch-MMR revealed MSI-H and a pathogenic *MSH2* germline mutation (c.942+3A>T) in a state of copy neutral LOH. Interestingly, this tumor also contained a pathogenic *MSH2* somatic mutation (R711^*^) at a lower frequency, suggesting this mutation was acquired as the tumor evolved (Figure 2B). In both cases, the biallelic inactivation of the MMR gene is likely responsible for MMR deficiency. The TumorNext-Lynch-MMR results corroborated the IHC data for both cases and are consistent with Lynch syndrome.

Another common scenario for suspected Lynch patients is IHC absent and/or MSI-H with no germline mutations detected in MMR genes. These results may create a clinical management quandary and patients are often advised to follow rigorous Lynch syndrome cancer screening protocols as Lynch syndrome cannot be ruled out. These discordant cases can be resolved by the detection of one somatic mutation in the context of LOH or two (or more) biallelic somatic mutations, which will rule out Lynch syndrome and negate the need for surveillance [18, 43]. The former scenario is illustrated by Patient 3, a 48 year old female with a history of adenocarcinoma of the right colon. IHC revealed absence of MLH1 and PMS2 protein and presence of MSH2 and MSH6 protein. TumorNext-Lynch-MMR revealed one somatic *MLH1* pathogenic mutation (c.461delA) in copy neutral LOH (Figure 2C), which explained the IHC results. The later scenario is illustrated by Patient 4, who presented with endometrial adenocarcinoma and absence of PMS2 protein and presence of MSH2, MSH6 and MLH1 protein on IHC. This patient had a family history of cancer, however, only somatic mutations were detected in *PMS2*; p.Q288^*^ (pathogenic mutation) and p.H701R (variant, likely pathogenic) (Figure 2D). In both of these cases, the somatic mutations explained the IHC results and are likely the cause of MMR deficiency.

Genetic testing for germline disorders, such as hereditary breast and ovarian cancer and Lynch syndrome, has become commonplace and the majority of variants detected can be classified as either benign or pathogenic. However, some variants detected are classified as variants of uncertain significance (VUS) [44]. The co-occurrence of a germline VUS with a somatic mutation or in a state of LOH may allow reclassification of the VUS to a variant that is likely pathogenic (VLP) when factored in with additional evidence (i.e. immunohistochemistry, family history, *in silico* modeling [45–47], protein structure analysis and variant database query), which increases diagnostic yield. We observed two such cases with the limited number of specimens received. The first case involved patient 5, who had a history of uterine serous adenocarcinoma and a germline VUS in *MSH6* (p.R976C) that was also detected in her mother. Initial tumor analysis showed MSI-H and absence of MSH6 protein on IHC. TumorNext-Lynch-MMR revealed a somatic pathogenic mutation (p.E118^*^) in *MSH6* (Figure 2E). The co-occurrence of the truncating mutation with p.R976C in *MSH6* in addition to the loss of MSH6 protein expression (factored in with family history, *in silico modeling*, etc.) provided evidence to upgrade this VUS to a likely pathogenic variant and diagnosis was changed to Lynch syndrome. A second case involved patient 6, who had clear cell carcinoma of the uterus and a germline VUS in *MSH6* (p.T767I). Tumor histology showed absence of MSH6 protein and presence of protein for MLH1, MSH2 and PMS2 on IHC. TumorNext-Lynch-MMR revealed a somatic pathogenic mutation (c.3261delC) in *MSH6* (Figure 2E). Again, the co-occurrence of this pathogenic mutation with p.T767I in *MSH6*, the loss of MSH6 protein expression and other factors provided evidence to upgrade this VUS to a likely pathogenic variant and the diagnosis was upgraded to Lynch syndrome.

NGS-based tumor profiling assays tend to require FFPE blocks with significant tissue as the DNA input requirements are typically 500 ng to 1 ug. Biopsies can pose a challenge for NGS assays as tissue may be limited, however, they are typically the standard specimen type for colorectal cancers submitted for traditional MMR testing (i.e. IHC and/or MSI) and there is value in analyzing DNA from the same tissue specimen that had been previously tested due to tumor heterogeneity. We have received several biopsies and have been successful in extracting sufficient amounts of DNA for analysis with TumorNext-Lynch-MMR. For example, a biopsy from patient 7, a 77 year old male with invasive adenocarcinoma of the colon, showed absence of protein for MLH1 and PMS2 on IHC and MSI-H. TumorNext-Lynch-MMR revealed a somatic pathogenic mutation in *MSH6* (c.3261dupC) and a second somatic pathogenic mutation in *MLH1* (p.E679^*^) in copy neutral LOH (Figure 2F). This patient also tested positive for the KRAS G12D mutation.

Hypermethylation of the *MLH1* promoter has been shown to cause MSI and be a cause of mismatch repair deficiency [48], so a methylation-specific multiplex ligation probe amplification (MLPA) assay from MRC Holland (Product no. ME011-B3/Amsterdam, the Netherlands) was included as a test option (validation data not shown) [49]. Approximately 14% of positive TumorNext-Lynch-MMR cases were due to *MLH1* hypermethylation. This is illustrated in Patient 8, a 68 year old female with endometrioid adenocarcinoma of the uterus with absence of MLH1 and PMS2 and presence of MSH2 and MSH6 on IHC. TumorNext-Lynch-MMR revealed MSI-H and *MLH1* in a state of LOH, but no germline or somatic mutations. MLPA detected *MLH1* promoter hypermethylation. These results are consistent with the lack of MLH1 and PMS2 protein expression.

In 2012, the University of Washington introduced ColoSeq™, an NGS-based test to specifically test for hereditary colon cancer [50]. ColoSeq™ Tumor was launched later, but is a standalone test for tumor tissue only. TumorNext-Lynch-MMR is a unique assay since it simultaneously detects and differentiates somatic and germline mutations as tumor and germline DNA are analyzed in parallel. The test is able to resolve discordant cases and accurately classify variants, which increases diagnostic yield. The process of analyzing both blood and tumor may also serve as confirmation of the presence of a germline mutation. The test has produced conclusive results in the majority of clinical specimens received and has identified mutations missed by other labs. One such sample involved patient 8, a 48 year old male CRC patient with a strong family history of colon, breast and skin cancer. Tumor histology showed absent protein expression for MSH2 and MSH6. Prior germline testing offered by a different lab did not detect any inherited mutations in *MSH2* or *MSH6*, so TumorNext-Lynch-MMR was ordered to test for somatic mutations that may be drivers of disease. TumorNext-Lynch-MMR revealed a germline *MSH2* founder mutation that resulted in an inversion of exons 1 – 7 (Boland mutation), a pathogenic somatic *MSH2* mutation (p.R389^*^), a pathogenic somatic *MSH6* mutation (c.3261delC) and somatic variant of unknown significance in *MSH6* (c.2561_2563delAGA) (Figure 2G). A diagnosis of Lynch Syndrome was made for this patient.

Ambry has analyzed patient samples from over 150 different institutions with TumorNext-Lynch-MMR and approximately 60% of the cases contained double somatic mutations in one of the five Lynch genes. If these patients already had germline testing, TumorNext-Lynch-MMR would not be reimbursed regardless of the fact that somatic testing provided the final diagnosis. These results highlight the problem with the logic behind new reimbursement policies for genetic testing. TumorNext-Lynch-MMR can serve as a model for new testing guidelines where genes are tested from both tumor tissue and blood. The suspected Lynch patients with double somatic mutations may no longer require surveillance in the form of annual or biennial colonoscopies, which is a significant cost savings for insurers.

## MATERIALS AND METHODS

### Validation samples

Genomic DNA was isolated from both FFPE tissue and peripheral blood from primarily colon and endometrial tumor specimens previously characterized on TumorNext (an NGS-based tumor profiling assay targeting 142 genes that are frequently mutated in somatic and/or germline cancers)[24], the Affymetrix OncoScan array and Promega MSI Analysis System [51]. The TumorNext-Lynch-MMR assay analyzes both germline and somatic mutations, LOH in the 5 Lynch genes and MSI. A variety of validation samples were selected as positive and negative controls based on the assessed feature. All FFPE specimens were reviewed by a pathologist and contained ≥20% tumor cellularity.

### NGS library and sample preparation

The TumorNext-Lynch-MMR assay was designed to analyze DNA isolated from both blood and tumor. A custom panel was designed to analyze 508 exons, 81 introns (partial) and 13 UTR regions in 39 genes. Five genes are associated with Lynch syndrome, 27 genes are associated with colorectal cancer and 7 genes are common in solid tumors (Supplementary Table 1). In addition, probes for the 5 “gold standard” microsatellite instability markers used in the Promega MSI Analysis System (NR-21, BAT-26, BAT-25, NR-24 and MONO-27) were included in the panel. The panel is composed of biotinylated xGen Lockdown probes synthesized by Integrated DNA Technologies (IDT, Coralville, IA).

Briefly, 500 ng DNA was sheared to an average size of 250-400bp using sonication (Covaris, Woburn, MA). DNA fragment ends were repaired and phosphorylated using Klenow, T4 DNA Polymerase, and T4 Polynucleotide Kinase. An ‘A’ base was added to the 3’ end of the blunted fragments, followed by ligation of single-indexed NGS adapters via T-A mediated ligation. The library was PCR-amplified using 8 cycles, and 11 libraries were pooled together (98 ng per tumor sample library and 10.9 ng per matched control, each with a unique sample index) and incubated with the IDT xGen Lockdown probes for 16 hours at 65°C. Captured DNA was washed, eluted and PCR amplified using 10 cycles. The size and concentration of the amplified captured DNA library were determined using the Agilent TapeStation or Bioanalyzer (Agilent, Santa Clara, CA). Each capture of 11 libraries (11 tumor + 11 matched blood = 22 DNA specimens, each with a unique sample index) was loaded onto one lane for sequencing on one flowcell of the Illumina HiSeq2500 (02-240nn-PTM). Samples previously characterized on TumorNext were prepared in an identical manner with a different IDT xGen Lockdown probe library.

### Data analysis

Demultiplexing by barcode and sequence quality filtering was done in the Illumina Consensus Assessment of Sequence and Variation (CASAVA) software (v.1.8.2, Illumina, Hayward, CA). A custom bioinformatics pipeline was developed to perform paired analysis of tumor and germline DNA. Briefly, FASTQ files from CASAVA were aligned to the hg19 version of the human genome using Novoalign V3.02.07. Next, paired-sample analysis was performed using VarScan2 (v2.3.8). For both SNP and indel calling by VarScan2, the minimum variant frequency was set to 1% and the minimum coverage in tumor and normal was set to 6x and 4x respectively. Optimized variant calling filters were set at a read coverage of ≥100x for tumor DNA and ≥10x for matched control. Paired normal samples were also analyzed using a custom bioinformatics pipeline that utilizes Novoalign V3.02.07 to align FASTQ reads to a reference sequence (hg19) and GATK (V3.2.2) to generate variants and no/low coverage reports. Germline variants were filtered using a Q score of ≥30, coverage of ≥10x, het ratio of ≥10% and filtered out if determined to be a sequencing artifact or common polymorphism utilizing population frequency data from multiple sources including NCBI dbSNP, NHLBI Exome Sequencing Project (ESP), 1000 Genomes, and internal Ambry data. Known causative variants outside reportable range are also protected from filtering. For quality control, the pipeline generates coverage metrics including: 1) number of total read pairs, 2) % of mapped read pairs, 3) % of PCR duplicates, 4) number of on-target read-pairs, 5) average coverage in target region, 6) target specificity and 7) % of bases at ≥10x, ≥20x, ≥50x, ≥100x, ≥200x, ≥500x, and ≥1000x.

The TumorNext pipeline was designed to achieve maximum sensitivity in detecting somatic variants in tumor samples whether matched control samples are available or not. In tumor-normal analysis mode, we applied Varscan2 (v2.3.6), a highly sensitive, heuristic based algorithm to detect somatic variants at low as 3% frequency. Current efforts are focused on developing custom filters to remove low confidence calls with evidence from literature and public repositories.

Germline vcf files were annotated using software developed in-house. The pipeline generates reports for variants detected and no- and low-coverage regions (nolocos). The Ambry NGS pipeline filters out variants with a Q score ≤30 and a het ratio of <10%. For germline DNA, noloco regions are generated for areas with <20X coverage in panels. Samples with greater than 10 nolocos are failed. Loss of heterozygosity was determined for somatic specimens by comparing primarily intronic allele frequencies between tumor and normal samples.

Germline variant reports generated from the Ambry NGS pipeline are converted to an AVA input format for upload. Samples are classified into several categories and filtered out if determined to be a polymorphism (utilizing population frequency data from multiple sources including NCBI dbSNP, NHLBI Exome Sequencing Project (ESP), 1000 Genomes, and an internal Ambry database) and/or outside of the analytical range. Alterations with likely clinical relevance are verified by Sanger sequencing.

### OncoScan

The OncoScan workflow is based on the hybridization of MIPs to FFPE DNA samples and subsequent circularization, amplification and labeling. The labeled MIPs are hybridized to the OncoScan array, washed and scanned. The assays were set up according to the OncoScan sample preparation manual (P/N 703175 Rev. 1) using DNA isolated from FFPE tumor specimens using the Qiagen GeneRead FFPE DNA extraction kit (Qiagen, Santa Clarita, CA). Briefly, DNA samples are normalized to 12 ng/μL, mixed with MIPs and incubated overnight to anneal (16-18 hours). Next, each reaction was divided equally into A and B reactions and “Gap Fill” master mix was added with either AT dNTPs (A reaction) or GC dNTPs (B reaction) and incubated. Following the “Gap Fill” reaction, exonuclease was added to remove unligated probes and genomic DNA. Next, MIPs were linearized with a restriction enzyme and PCR amplified (PCR 1). Reactions were taken through a second round of amplification (PCR 2) and subsequently digested with HaeIII restriction enzyme. The digested products were hybridized to the OncoScan Array for 16-18hrs. Arrays were stained and washed using the GeneChip® Fluidics Station 450 and loaded on the GeneChip® Scanner 3000 7G (Affymetrix, Santa Clara, CA) where fluorescence intensity was scanned to generate array images (DAT files). Next, array fluorescence intensity data (CEL) files were generated and used to produce OSCHP-TuScan files with the OncoScan® Console software version 1.1 using the reference files OncoScan.FFPE.n33.r1.REF_MODEL for CNVs and OncoScan.FFPE.n33.r1.SOM_REF_MODEL for SNPs.

The TuScan algorithm is based on the ASCAT (allele-specific copy number analysis of tumors) algorithm, which determines allele specific copy number and simultaneously estimates and adjusts for both percent tumor and ploidy [52]. It provides copy number in log2 and linear scale, which can be viewed in Nexus Express for OncoScan. OncoScan uses the logR and BAF to determine copy number. The logR ratio is the logged ratio of observed probe intensity to the expected intensity – any deviations from zero indicate copy number change. BAF allows detection of allelic imbalance. A value near 0.5 indicates a heterozygous genotype (AB), whereas 0 and 1 indicate a homozygous genotype (AA and BB) – in a normal diploid sample, there is a mix of AA, AB and BB genotypes. Deletions, copy neutral loss of heterozygosity, imbalanced amplifications and mosaic samples exhibit altered BAF plots. Unlike algorithms that use uniform thresholds, TuScan can detect CNVs when only present in a minority of cells as the algorithm determines what deviations from logR and BAF are consistent with the percent tumor and ploidy of the sample.

### Microsatellite instability detection

The Promega MSI Analysis System (Promega, Madison, WI) was used as the gold standard to compare the NGS approach for MSI analysis. The kit analyzes the mononucleotide repeat regions NR-21, BAT-26, BAT-25, NR-24 and MONO-27 to check for deletions or insertions. The kit also analyzes the pentanucleotide markers Penta C and Penta D for quality control (i.e. tumor/normal sample matching). DNA from both tumor and matched normal blood were PCR amplified to generate fluorescently labeled amplicons of the repeat regions. The amplicons were separated based on size using capillary gel electrophoresis on an ABI 3730xl DNA Analyzer (Applied Biosystems, Carlsbad, CA). Fragment analysis was performed using GeneMapper software, which compares the allelic patterns of the matched normal and tumor samples. Samples with alterations in the length of the repeat region (i.e. microsatellite region) due to deletion or insertion in more than or equal to 2 out of the 5 mononucleotide repeat markers are classified as MSI-High status. If zero or one markers are altered in length compared to the matched normal, the sample was classified as stable (i.e. normal).

### Methylation specific – multiplex ligation-dependent probe amplification

The *MLH1* promoter methylation status was determined by using the *MLH1* Methylation Specific – Multiplex Ligation-dependent Probe Amplification (MS-MLPA) kit (ME011-B3, MRC Holland, Netherlands), which contains probes covering the *MLH1* promoter at five CpG islands. Briefly, genomic DNA is denatured and incubated with MLPA probes for 16 hours to facilitate probe hybridization. The sample was divided into two wells; one well was treated with the standard MLPA Ligase 65 to ligate the probes together and the second well was treated with Ligase 65 and *HhaI* restriction enzyme. The *HhaI* enzyme is methylation sensitive and will not digest DNA that is methylated. Reaction mixes were then PCR amplified with universal FAM tagged primers and the products were resolved on the ABI 3730xl. MS-MLPA data are analyzed using Coffalyser software, which compares the amplicon signal intensity between the digested and undigested portions of the same sample and reports a ratio. A ratio of 1 indicates there is no methylation (i.e. there is no difference between the *HhaI* treated and untreated DNA). A ratio of 0.3 or higher (for one CpG island) or 0.15 or higher (for multiple CpG islands) indicates DNA methylation.

## SUPPLEMENTARY MATERIALS TABLES




