# Supplementary material for: TumorNext-Lynch-MMR: a comprehensive next generation sequencing assay for the detection of germline and somatic mutations in genes associated with mismatch repair deficiency and Lynch syndrome
Source: Oncotarget. 2018 Apr 17;9(29):20304–22. doi: 10.18632/oncotarget.24854 (PMC5945525; doi:10.18632/oncotarget.24854)
Supplement: Supplementary file 1 [file oncotarget-09-20304-s001.pdf]

TumorNext-Lynch-MMR: a comprehensive next generation sequencing assay for the detection of germline and somatic mutations in genes associated with mismatch repair deficiency and Lynch syndrome

SUPPLEMENTARY MATERIALS

Supplementary Table 1: Genes Targeted in TumorNext-Lynch-MMR

AKT1	BRAF	CDKN2A	FBXW7	MLH1	MYC	PMS2	SMAD4	BAT-25*
APC	BUB1	CHEK2	GALNT12	MSH2	NRAS	POLD1	STK11	BAT-26*
AXIN2	BUB3	CTNNA1	GREM1	MSH3	NTHL1	POLE	TERT	MONO-27*
BLM	CCND1	EPCAM	GRID1	MSH6	PDGFRA	PTEN	TP53	NR-21*
BMPR1A	CDH1	ERBB2	KRAS	MUTYH	PIK3CA	RPS20	Penta C*	NR-24*

*MSI targets

Supplementary Table 2: Results of Accuracy Study for Somatic Mutation Detection for TumorNext-Lynch-MMR

See Supplementary File 1

Supplementary Table 3: Results of Accuracy Study for Germline Variant Detection for TumorNext-Lynch-MMR

See Supplementary File 1

Supplementary Table 4: Genomic regions in TumorNext-Lynch-MMR panel used for MSI analysis

Chromosome	Start Position	Stop Position	Repeat Type/Length	Gene(s)/Marker name	Region
chr1	239881909	239881918	(T)10	CHRM3, CHRM3-AS2	Intronic
chr2	16086265	16086274	(T)10	MYCN	3UTR
chr2	47600582	47600591	(T)10	EPCAM	Intronic
chr2	47614204	47614219	(T)16	EPCAM	Intergenic (37 bps from last exon of EPCAM)
chr2	47635524	47635536	(T)13	MSH2	Intronic
chr2	47702452	47702470	(T)19	MSH2	Intronic
chr2	48005091	48005100	(T)10	MSH6	Intergenic (5,130 bps from first exon of MSH6)
chr2	48032741	48032753	(T)13	MSH6	Intronic
chr2	48033891	48033908	(T)18	MSH6	Intronic
chr2	111406969	111406983	(A)15	BUB1	Intronic
chr2	215657183	215657198	(A)16	BRAD1	Intronic
chr4	153268228	153268241	(A)14	FBXW7	Intronic
chr5	80088494	80088503	(T)10	MSH3	Intronic
chr5	112111310	112111322	(A)13	APC	Intronic
chr7	6037058	6037074	(A)17	PMS2	Intronic
chr7	140434575	140434585	(A)11	BRAF	Intronic
chr7	140434587	140434596	(A)10	BRAF	Intronic
chr10	87359477	87359487	(T)11	GRID1-AS1, GRID1	Intronic, 3UTR
chr10	89720634	89720648	(T)15	PTEN	Intronic
chr10	89725294	89725304	(T)11	PTEN	3UTR
chr11	94212931	94212941	(A)11	MRE11	Intronic
chr11	108114662	108114676	(T)15	ATM	Intronic
chr11	108121411	108121425	(T)15	ATM	Intronic
chr11	108141956	108141970	(T)15	ATM	Intronic
chr11	108188267	108188279	(T)13	ATM, C11orf65	Intronic, Intronic
chr11	108195977	108195995	(T)19	ATM, C11orf65	Intronic, Intronic
chr12	133237754	133237767	(A)14	POLE	Intronic
chr13	32893198	32893207	(T)10	BRCA2	Intronic
chr13	32905220	32905231	(T)12	BRCA2	Intronic
chr13	32907536	32907546	(T)11	BRCA2	Intronic
chr15	91303326	91303337	(T)12	BLM	5UTR in isoforom NM_001287248, Intronic in other isoform(s)
chr15	91307366	91307375	(T)10	BLM	Intronic
chr16	23615043	23615056	(A)14	PALB2	Intronic
chr17	7577679	7577694	(T)16	TP53	Intronic
chr17	41256075	41256086	(A)12	BRCA1	Intronic
chr17	41256088	41256097	(A)10	BRCA1	Intronic
chr17	59857600	59857610	(A)11	BRIP1	Intronic
chr18	48573269	48573280	(T)12	SMAD4	Intronic
chr18	48584856	48584871	(T)16	SMAD4	Intronic
chr19	1206797	1206806	(T)10	STK11	5UTR
chr2	39536690	39536716	(T)27	MAP4K3 (MONO-27)	Intronic
chr2	95849362	95849384	(T)23	ZNF2 (NR-24)	3UTR
chr14	23652347	23652367	(A)21	SLC7A8 (NR-21)	5UTR
chr4	55598212	55598236	(T)25	KIT (BAT-25)	Intronic
chr2	47641560	47641586	(A)27	MSH2 (BAT-26)	Intronic

Supplemental Table 5: Inter-assay reproducibility results for somatic variants

See Supplementary File 1

Supplementary Table 6: Inter-assay reproducibility results for germline variants

See Supplementary File 1

Supplementary Table 7: Inter-assay reproducibility results for MSI analysis

Sample	**Validation 1MSI Status**	**Validation 2MSI Status**	**Validation 3MSI Status**
BR_14_97_07_01_T	Stable	Stable	Stable
BR-14-131-05-01-T	Stable	Stable	Stable
BR-13-162-05-01T	Stable	Stable	Stable
RD_009	Stable	Stable	Stable
BR-14-283-05-01T	Stable	Stable	Stable
BR-13-184-05-01T	Stable	Stable	Stable
BR-13-116-05-01T	Stable	Stable	Stable
BR-12-110-T	High	High	High
BR-14-239-05-01-T	Stable	Stable	Stable
BR-11-93-T	Stable	Stable	Stable

Supplementary Table 8: Inter-assay reproducibility for determining LOH using targeted capture of select introns

Sample	Gene	Validation Run 1	Validation Run 2	Validation Run 3
BR-11-71	MSH2	No	No	No
	PMS2	No	No	No
	MSH6	No	No	No
	MLH1	No	No	No
	EPCAM	No	No	No
BR-13-102	MSH2	No	No	No
	PMS2	No	No	No
	MSH6	No	No	No
	MLH1	No	No	No
	EPCAM	No	No	No
BR-13-191	MSH2	No	No	No
	PMS2	No	No	No
	MSH6	No	No	No
	MLH1	No	No	No
	EPCAM	No	No	No
BR-15-90	MSH2	No	No	No
	PMS2	No	No	No
	MSH6	No	No	No
	MLH1	Yes	Yes	Yes
	EPCAM	No	No	No
BR13-116	MSH2	No	No	No
	PMS2	No	No	No
	MSH6	No	No	No
	MLH1	Yes	Yes	Yes
	EPCAM	No	No	No
BR13-81	MSH2	No	No	No
	PMS2	No	No	No
	MSH6	No	No	No
	MLH1	Yes	Yes	Yes
	EPCAM	No	No	No
BR13-97	MSH2	No	No	No
	PMS2	No	No	No
	MSH6	No	No	No
	MLH1	Yes	Yes	Yes
	EPCAM	No	No	No
BR14_26	MSH2	No	No	No
	PMS2	Yes	Yes	Yes
	MSH6	No	No	No
	MLH1	No	No	No
	EPCAM	No	No	No

Supplementary Table 9: Intra-assay reproducibility study for somatic variants

Sample	Mutation	Replicate 1	Replicate 2	Replicate 3
BR-13-184-05-01T	KRAS NM_033360 c.38G>A p.G13D	74.3%	72.5%	70.7%
BR-13-184-05-01T	TP53 NM_000546 c.556delG p.D186Mfs*61	76.9%	69.1%	71.0%
BR-13-184-05-01T	APC NM_000038 c.3907C>T p.Q1303*	67.2%	69.7%	69.1%

Supplementary Table 10: Intra-assay reproducibility study for germline variants

Sample	Mutation	Replicate 1 Frequency/depth of coverage	Replicate 2 Frequency/depth of coverage	Replicate 3 Frequency/depth of coverage
BR-13-184-05-01T	POLD1 NM_002691 c.1485C>T p.T495T	49.69% 322	44.25% 287	47.04% 372
BR-13-184-05-01T	POLE NM_006231 c.6252A>G p.S2084S	48.61% 323	44.44% 279	43.95% 314
BR-13-184-05-01T	POLE NM_006231 c.3156G>A p.T1052T	51.27% 355	47.7% 306	47.94% 389
BR-13-184-05-01T	PMS2 NM_000535 c.1621A>G p.E541E	100% 318	100% 247	100% 336
BR-13-184-05-01T	PMS2 NM_000535 c.1569C>G p.S523S	45.03% 382	48.53% 308	47.63% 402
BR-13-184-05-01T	MLH1 NM_000249 c.655A>G p.I219V	48.93% 280	49.36% 236	52.17% 276
BR-13-184-05-01T	PMS2 NM_000535 c.780C>G p.S260S	99.50% 203	100% 143	100% 210
BR-13-184-05-01T	CDH1 NM_004360 c.2076T>C p.A692A	48.65% 333	47.02% 285	47.49% 358
BR-13-184-05-01T	PMS2 NM_000535 c.1408C>T p.P470S	100% 362	100% 296	100% 375
BR-13-184-05-01T	POLE NM_006231 c.4530A>G p.A1510A	49.37% 319	46.21% 290	49.87% 373
BR-13-184-05-01T	PMS2 NM_000535 c.2570G>C p.G857A	38.64% 44	22.22% 27	20% 35

Supplementary Table 11: Intra-assay reproducibility results for MSI analysis

Sample	Replicate 1	Replicate 2	Replicate 3
BR-13-184-05-01T	Stable	Stable	Stable

Supplementary Table 12: Intra-assay reproducibility for determining LOH using targeted capture of select introns

Sample	Gene	Replicate 1 (161209_ D00314_0676_ BHHWWJBCXX – lane 1)	Replicate 3 (161209_ D00314_0676_ BHHWWJBCXX – lane 1)	Replicate 3 (161209_ D00314_0676_ BHHWWJBCXX – lane 1)
BR15_168	MSH2	Yes	Yes	Yes
	PMS2	Yes*	No	No
	MSH6	Yes	Yes	Yes
	MLH1	No	No	No
	EPCAM	Yes	Yes	Yes

*Allelelic imbalance due to CN = 3. Low frequency LOH detected in replicate 1, but not in replicates 2 and 3.
