# Supplementary material for: TumorNext-Lynch-MMR: a comprehensive next generation sequencing assay for the detection of germline and somatic mutations in genes associated with mismatch repair deficiency and Lynch syndrome
Source: Oncotarget. 2018 Apr 17;9(29):20304–22. doi: 10.18632/oncotarget.24854 (PMC5945525; doi:10.18632/oncotarget.24854)
Supplement: Supplementary file 2 [file oncotarget-09-20304-s002.docx]

| **Supplemental Table 2: Results of Accuracy Study for Somatic Mutation Detection for TumorNext-Lynch-MMR** | | | | |
| --- | --- | --- | --- | --- |
| **Sample ID** | **Mutation** | **Previous Mutation Frequency** | **Accuracy Mutation Frequency** | Comments |
| BR-11-49-396-4T | BRAF NM_004333 c.1799T>A p.V600E | 24.63% | 27.75% |  |
| BR-11-71-T | APC NM_000038 c.5296G>A p.D1766N | 6.81% | 8.05% |  |
|  | TP53 NM_000546 c.578A>T p.H193L | 53.09% | 55.37% |  |
| BR-11-93-T | TP53 NM_000546 c.404G>T p.C135F | 24.10% | 25.69% |  |
| BR-12-110-T | MSH6 NM_000179 c.3261delC p.F1088Sfs*2 | 8.06% | 5.38% |  |
|  | FBXW7 NM_033632 c.585-5delT NULL | 30.15% | 33.59% |  |
|  | BRAF NM_004333 c.2128-6_2128-5delTT NULL | 19.05% | 14.53% |  |
|  | BRAF NM_004333 c.1208delC p.P403Lfs*8 | 26.73% | 24.88% |  |
|  | PTEN NM_000314 c.800delA p.K267Rfs*9 | 52.19% | 45.66% |  |
|  | KRAS NM_033360 c.35G>A p.G12D | 48.74% | 49.26% |  |
| BR-12-11-1041-3T | FBXW7 NM_033632 c.2065C>T p.R689W | 20% | 32.46% |  |
|  | APC NM_000038 c.1690C>T p.R564* | 16.92% | 29.49% |  |
|  | APC NM_000038 c.4348C>T p.R1450* | 17.82% | 29.90% |  |
| BR-12-15-T | KRAS NM_033360 c.35G>A p.G12D | 6.45% | 6.12% |  |
| BR-12-30-T | MYCN NM_005378 c.134delC p.P45Rfs*86 | 25.91% | 18.34% |  |
|  | MYCN NM_005378 c.550G>A p.A184T | 22.71% | 11.65% |  |
|  | MSH6 NM_000179 c.3261dupC p.F1088Lfs*5 | 18.45% | 10.81% |  |
|  | FBXW7 NM_033632 c.585-7_585-5delTTT NULL | 24.48% | 22.20% |  |
|  | BRAF NM_004333 c.1799T>A p.V600E | 20.22% | 12.98% |  |
|  | AKT1 NM_005163 c.303C>T p.T101T | 15.48% | 13.14% |  |
|  | CDH1 NM_004360 c.1422C>T p.T474T | 22.26% | 14.94% |  |
|  | STK11 NM_000455 c.842delC p.P281Rfs*6 | 21.85% | 12.41% |  |
|  | APC NM_000038 c.7212G>A p.M2404I | 7.50% | 1.49% | Filtered out due to <5% |
| BR-13-102-T | CDH1 NM_004360 c.683dupA p.Y228* | 51.46% | 43.18% |  |
| BR-13-162-05-01T | MSH6 NM_000179 c.3881G>T p.C1294F | 9.01% | 10.32% |  |
|  | APC NM_000038 c.621C>A p.C207* | 8.60% | 11.11% |  |
|  | APC NM_000038 c.4285C>T p.Q1429* | 10.57% | 12.63% |  |
|  | BRAF NM_004333 c.1237G>A p.V413M | 9.87% | 10.35% |  |
|  | TP53 NM_000546 c.817C>T p.R273C | 13.03% | 15.40% |  |
| BR-13-163-T | FBXW7 NM_033632 c.1393C>T p.R465C | 20.69% | 22.16% |  |
|  | APC NM_000038 c.994C>T p.R332* | 19.54% | 17.88% |  |
|  | APC NM_000038 c.2222delA p.N741Ifs*20 | 20.08% | 17.54% |  |
|  | APC NM_000038 c.4666dupA p.T1556Nfs*3 | 18.71% | 19.64% |  |
|  | KRAS NM_033360 c.35G>T p.G12V | 13.81% | 19.72% |  |
|  | TP53 NM_000546 c.818G>A p.R273H | 26.14% | 32.01% |  |
| BR-13-184-05-01T | APC NM_000038 c.3907C>T p.Q1303* | 55.04% | 71.39% |  |
|  | KRAS NM_033360 c.38G>A p.G13D | 60.13% | 77.27% |  |
|  | TP53 NM_000546 c.556delG p.D186Mfs*61 | 57.49% | 67.92% |  |
| BR-13-191-05-01T | TP53 NM_000546 c.818G>A p. R273H | 29% | 26% |  |
|  | BRAF NM_004333 c.1799T>A p.V600E | 28% | 30% |  |
| BR-13-25-05-01T | APC NM_000038 c.2607delT p.P870Qfs*46 | 34.37% | 42.32% |  |
|  | APC NM_000038 c.3872dupA p.T1292Dfs*9 | 13.79% | 17.76% |  |
|  | ATM NM_000051 c.1034T>C p.L345S | 17.71% | 25.94% |  |
|  | KRAS NM_033360 c.35G>A p.G12D | 19.49% | 30.67% |  |
|  | TP53 NM_000546 c.818G>A p.R273H | 28.37% | 42.06% |  |
| BR-13-29-05-01-T | MSH2 NM_000251 c.909T>C p.D303D | 15.75% | 17.60% |  |
|  | MSH2 NM_000251 c.*4delA NULL | 14.76% | 16.65% |  |
|  | FBXW7 NM_033632 c.1177C>T p.R393* | 20.14% | 23.74% |  |
|  | PTEN NM_000314 c.253G>T p.V85F | 19.47% | 20.41% |  |
|  | PTEN NM_000314 c.469dupG p.E157Gfs*23 | 20.59% | 20.89% |  |
|  | KRAS NM_033360 c.181C>A p.Q61K | 17.03% | 18.73% |  |
|  | KRAS NM_033360 c.180T>A p.G60G | 16.99% | 18.48% |  |
|  | TP53 NM_000546 c.817C>T p.R273C | 19.63% | 23.41% |  |
|  | TP53 NM_000546 c.216delC p.V73Wfs*50 | 23.02% | 22.83% |  |
| BR-14-13-05-01-T | PIK3CA NM_006218 c.1634A>C p.E545A | 5.85% | 8.02% |  |
|  | PIK3CA NM_006218 c.2908G>A p.E970K | 5.21% | 8.93% |  |
|  | APC NM_000038 c.637C>T p.R213* | 7.88% | 9.52% |  |
|  | APC NM_000038 c.694C>T p.R232* | 8.18% | 10.63% |  |
| BR-14-131-05-01-T | APC NM_000038 c.3340C>T p.R1114* | 32.45% | 32.56% |  |
|  | APC NM_000038 c.3925G>T p.E1309* | 17.49% | 16.68% |  |
|  | TP53 NM_000546 c.772G>T p.E258* | 46.61% | 47.07% |  |
| BR-14-194-05-01-T | APC NM_000038 c.2626C>T p.R876* | 21.03% | 23.67% |  |
|  | APC NM_000038 c.4666dupA p.T1556Nfs*3 | 43.32% | 43.01% |  |
|  | KRAS NM_033360 c.35G>A p.G12D | 32.98% | 38.43% |  |
|  | TP53 NM_000546 c.856G>A p.E286K | 25.35% | 25.09% |  |
|  | TP53 NM_000546 c.637C>T p.R213* | 51.84% | 50.07% |  |
| BR-14-209-05-01-T | MSH6 NM_000179 c.3261delC p.F1088Sfs*2 | 15.21% | 17.09% |  |
|  | MLH1 NM_000249 c.1489delC p.R497Gfs*11 | 17.25% | 13.78% |  |
|  | PIK3CA NM_006218 c.3140A>G p.H1047R | 11.04% | 11.37% |  |
|  | APC NM_000038 c.4385delA p.K1462Rfs*11 | 12.24% | 14.49% |  |
|  | KRAS NM_033360 c.34G>A p.G12S | 10.92% | 12.92% |  |
|  | CDH1 NM_004360 c.1199A>T p.D400V | 6.01% | 7.24% |  |
| BR-14-239-05-01-T | PIK3CA NM_006218 c.1633G>A p.E545K | 8.69% | 7.44% |  |
|  | APC NM_000038 c.1935_1951del17 p.I646Pfs*22 | 8.62% | 15.88% |  |
|  | KRAS NM_033360 c.35G>T p.G12V | 39.14% | 47.11% |  |
|  | TP53 NM_000546 c.713G>T p.C238F | 43.47% | 38.48% |  |
| BR-14-43-05-01-T | APC NM_000038 c.1690C>T p.R564* | 5.39% | 5.29% |  |
|  | APC NM_000038 c.3911delT p.I1304Kfs*4 | 5.11% | 5.81% |  |
|  | TP53 NM_000546 c.871A>T p.K291* | 11.92% | 13.60% |  |
|  | TP53 NM_000546 c.869G>T p.R290L | 12.05% | 13.90% |  |
| BR-14-48-05-01-T | APC NM_000038 c.2377C>T p.Q793* | 19.61% | 19.37% |  |
|  | APC NM_000038 c.4348C>T p.R1450* | 18.31% | 17.32% |  |
|  | TP53 NM_000546 c.872dupA p.K292Efs*14 | 42.26% | 48.18% |  |
| BR-14-88-05-01-T | NRAS NM_002524 c.182A>G p.Q61R | 17.26% | 18.12% |  |
|  | BRAF NM_004333 c.1447A>G p.K483E | 12.68% | 14.24% |  |
|  | TP53 NM_000546 c.376-1G>A NULL | 18.33% | 20.20% |  |
| BR_13_170_05_01_T | SMAD4_NM_005359_c.1392C>A_p.A464A | 24.04% | 29.32% |  |
|  | PTEN_NM_000314_c.821G>A_p.W274* | 73.23% | 70.10% |  |
| BR_13_187_05_02T_T | MLH1_NM_000249_c.1039-5T>G_ | 5.25% | 8.18% |  |
|  | PIK3CA_NM_006218_c.1624G>A_p.E542K | 49.76% | 49.28% |  |
| BR_14_138_05_02_T | APC_NM_000038_c.4240delG_p.V1414* | 33.04% | 33.66% |  |
|  | APC_NM_000038_c.2962G>T_p.E988* | 33.79% | 34.30% |  |
|  | SMAD4_NM_005359_c.1052A>T_p.D351V | 55.99% | 62.44% |  |
|  | KRAS_NM_033360_c.35G>T_p.G12V | 67.36% | 65.04% |  |
| BR_14_20_05_01T_T | PTEN_NM_000314_c.26_48del23_p.V9Afs*27 | 7.73% | 7.85% |  |
|  | PTEN_NM_000314_c.667A>T_p.K223* | 19.66% | 13.73% |  |
|  | PIK3CA_NM_006218_c.263G>A_p.R88Q | 15.27% | 13.19% |  |
|  | PTEN_NM_000314_c.48_49insAAC_p.Y16_Q17insN | 8.18% | 8.12% |  |
|  | PIK3CA_NM_006218_c.323G>A_p.R108H | 13.64% | 12.84% |  |
| BR_14_209_05_03_T | APC_NM_000038_c.4385delA_p.K1462Rfs*11 | 7.24% | 13.29% |  |
|  | MLH1_NM_000249_c.1489delC_p.R497Gfs*11 | 11.84% | 5.62% |  |
|  | PIK3CA_NM_006218_c.3140A>G_p.H1047R | 9.63% | 9.33% |  |
|  | MSH6_NM_000179_c.3261delC_p.F1088Sfs*2 | 11.08% | 12.50% |  |
|  | KRAS_NM_033360_c.34G>A_p.G12S | 11.78% | 14.75% |  |
| BR_14_209_05_03_T | KIT_NM_000222_c.2162A>G_p.Y721C | 11.79% | 0% | no coverage in region |
| BR_14_231_05_03T_T | APC_NM_000038_c.4093G>T_p.G1365C | 8.88% | 15.38% |  |
|  | TP53_NM_000546_c.456_457insGG_p.P153Gfs*18 | 64.08% | 68.31% |  |
|  | APC_NM_000038_c.3394G>T_p.E1132* | 34.60% | 38.24% |  |
|  | APC_NM_000038_c.3927_3931delAAAGA_p.E1309Dfs*4 | 35.39% | 34.09% |  |
| BR_14_240_05_01_T | PTEN_NM_000314_c.389G>A_p.R130Q | 32.15% | 34.15% |  |
|  | PIK3CA_NM_006218_c.1636C>A_p.Q546K | 11.86% | 12.38% |  |
| BR_14_248_05_02_T | KRAS_NM_033360_c.34G>T_p.G12C | 27.62% | 35% |  |
|  | TP53_NM_000546_c.722C>T_p.S241F | 34.96% | 33.04% |  |
| BR_14_253_05_02_T | CDKN2A_NM_000077_c.238C>T_p.R80* | 46.83% | 43.71% |  |
|  | KRAS_NM_033360_c.35G>A_p.G12D | 21.41% | 19.66% |  |
|  | FBXW7_NM_033632_c.1514G>A_p.R505H | 27.31% | 27.90% |  |
| BR_14_257_05_03_T | PTEN_NM_000314_c.760A>T_p.K254* | 29.50% | 26.83% |  |
|  | APC_NM_000038_c.4393_4394delAG_p.S1465Wfs*3 | 23.93% | 24.82% |  |
|  | APC_NM_000038_c.2804dupA_p.Y935* | 27.78% | 27.02% |  |
|  | KRAS_NM_033360_c.35G>T_p.G12V | 27.74% | 33.53% |  |
| BR_14_267_05_02_T | PTEN_NM_000314_c.511C>T_p.Q171* | 24.22% | 29.93% |  |
|  | PTEN_NM_000314_c.724G>T_p.E242* | 28.19% | 27.31% |  |
|  | CCND1_NM_053056_c.888dupA_ | 24.01% | 25.87% |  |
|  | CCND1_NM_053056_c.884_888delTCTGA_p.I295Rfs*57 | 22.61% | 24.55% |  |
| BR_14_293_05_01_T | APC_NM_000038_c.1495C>T_p.R499* | 29.09% | 23.88% |  |
|  | FBXW7_NM_033632_c.1435C>T_p.R479* | 51.72% | 50.51% |  |
|  | TP53_NM_000546_c.844C>T_p.R282W | 73.35% | 73.08% |  |
|  | APC_NM_000038_c.4189G>T_p.E1397* | 26.92% | 24.05% |  |
| BR_14_312_05_01_T | FBXW7_NM_033632_c.585-7_585-5delTTT_ | 26% | 26.98% |  |
|  | MSH2_NM_000251_c.942+17_942+29del13_ | 26.19% | 23.08% |  |
|  | TP53_NM_000546_c.902delC_p.P301Qfs*44 | 32.09% | 36.19% |  |
| BR_14_48_05_02T_T | APC_NM_000038_c.2377C>T_p.Q793* | 28.51% | 33.49% |  |
|  | APC_NM_000038_c.4348C>T_p.R1450* | 28.69% | 26.38% |  |
| BR_14_51_05_01_T | KRAS_NM_033360_c.35G>A_p.G12D | 9.31% | 12.33% |  |
|  | PTEN_NM_000314_c.35dupA_p.N12Kfs*32 | 12.01% | 13.91% |  |
|  | PTEN_NM_000314_c.955_958delACTT_p.T319* | 11.85% | 12.56% |  |
| BR_14_97_07_01_T | TP53_NM_000546_c.578A>G_p.H193R | 51.08% | 52.09% |  |
|  | APC_NM_000038_c.7849G>A_p.E2617K | 37.54% | 33.66% |  |
| BR_15_103_05_01_T | TP53_NM_000546_c.993+2T>C_ | 36.96% | 43.27% |  |
|  | PTEN_NM_000314_c.968delA_p.N323Mfs*21 | 34.75% | 34.75% |  |
|  | CDKN2A_NM_000077_c.304dupG_p.A102Gfs*18 | 38.16% | 40.64% |  |
|  | BRAF_NM_004333_c.1799T>A_p.V600E | 37.24% | 33.95% |  |
|  | FBXW7_NM_033632_c.1972C>T_p.R658* | 38.29% | 39.91% |  |
|  | MUTYH_NM_001128425_c.306C>T_p.S102S | 34.08% | 42.72% |  |
|  | ERBB2_NM_004448_c.1955C>T_p.T652M | 33.64% | 31.78% |  |
|  | FBXW7_NM_033632_c.585-7_585-5delTTT_ | 25.14% | 31.61% |  |
|  | PTEN_NM_000314_c.867delA_p.V290* | 35.71% | 38.50% |  |
| BR_15_12_05_01_T | PIK3CA_NM_006218_c.3140A>G_p.H1047R | 6.78% | 4.98% | Filtered out due to <5% |
|  | MSH6_NM_000179_c.3261delC_p.F1088Sfs*2 | 9.64% | 8.70% |  |
|  | BRAF_NM_004333_c.1799T>A_p.V600E | 21.14% | 26.09% |  |
|  | APC_NM_000038_c.2544dupA_p.D849Rfs*2 | 20.75% | 22.94% |  |
|  | APC_NM_000038_c.4348C>T_p.R1450* | 22.95% | 24.55% |  |
|  | FBXW7_NM_033632_c.2051G>A_p.C684Y | 7.62% | 5.56% |  |
| BR_15_168_05_01_T | TP53_NM_000546_c.637C>T_p.R213* | 21.99% | 26.46% |  |
|  | APC_NM_000038_c.646C>T_p.R216* | 17.32% | 20% |  |
|  | APC_NM_000038_c.3876_3879dupGACA_p.Q1294Dfs*8 | 18.51% | 18.50% |  |
| BR_15_23_05_01_T | APC_NM_000038_c.646C>T_p.R216* | 34.72% | 36.05% |  |
|  | TP53_NM_000546_c.481G>A_p.A161T | 38.02% | 39.49% |  |
|  | KRAS_NM_033360_c.38G>A_p.G13D | 33.74% | 36.59% |  |
| BR_15_23_05_01_T | MLH1_NM_000249_c.1039-5T>G_ | 5.85% | 0% | Coverage is only 98x |
| BR_15_37_05_01_T | TP53_NM_000546_c.817C>T_p.R273C | 35.46% | 34.01% |  |
|  | APC_NM_000038_c.4393_4394delAG_p.S1465Wfs*3 | 19.66% | 21.60% |  |
|  | MSH6_NM_000179_c.3261delC_p.F1088Sfs*2 | 24.01% | 28.66% |  |
|  | MSH2_NM_000251_c.1648A>T_p.K550* | 31.42% | 26.32% |  |
|  | TP53_NM_000546_c.475G>A_p.A159T | 28.40% | 29.71% |  |
|  | PTEN_NM_000314_c.697C>T_p.R233* | 27.80% | 25.74% |  |
|  | MSH2_NM_000251_c.942+14_942+29del16_ | 22.31% | 24.03% |  |
|  | APC_NM_000038_c.8384C>T_p.A2795V | 21.57% | 31.88% |  |
|  | PTEN_NM_000314_c.518G>A_p.R173H | 25.83% | 33.23% |  |
|  | KRAS_NM_033360_c.35G>A_p.G12D | 22.66% | 26.43% |  |
|  | APC_NM_000038_c.3313C>T_p.R1105W | 24.52% | 24.62% |  |
|  | APC_NM_000038_c.4234G>T_p.G1412* | 26.64% | 26.32% |  |
|  | STK11_NM_000455_c.1167C>T_p.A389A | 37.80% | 36.87% |  |
|  | APC_NM_000038_c.6049A>G_p.T2017A | 9.38% | 7.41% |  |
| BR_15_51_05_02_T | PTEN_NM_000314_c.85delT_p.Y29Ifs*25 | 50.09% | 54.84% |  |
|  | PIK3CA_NM_006218_c.1634A>C_p.E545A | 19.77% | 25.38% |  |
|  | KRAS_NM_033360_c.35G>T_p.G12V | 15.51% | 13.27% |  |
|  | MSH6_NM_000179_c.3261delC_p.F1088Sfs*2 | 11.06% | 4.67% | Filtered out due to <5% |
| BR_15_77_05_01_T | ERBB2_NM_004448_c.2264T>C_p.L755S | 11.88% | 13.90% |  |
|  | TP53_NM_000546_c.541C>T_p.R181C | 6.75% | 7.62% |  |
|  | MSH6_NM_000179_c.3312dupT_p.G1105Wfs*3 | 5.60% | 5.67% |  |
|  | ERBB2_NM_004448_c.2524G>A_p.V842I | 15.41% | 13.14% |  |
|  | FBXW7_NM_033632_c.1394G>A_p.R465H | 6.77% | 7.80% |  |
|  | MSH6_NM_000179_c.3209delG_p.G1070Vfs*9 | 5.81% | 6.50% |  |
|  | APC_NM_000038_c.1660C>T_p.R554* | 5.26% | 4.77% | Filtered out due to <5% |
|  | APC_NM_000038_c.4348C>T_p.R1450* | 5.21% | 4.62% | Filtered out due to <5% |
| BR_15_90_05_01_T | TP53_NM_000546_c.743G>A_p.R248Q | 30.73% | 36.63% |  |
|  | PIK3CA_NM_006218_c.1624G>A_p.E542K | 23.96% | 27.49% |  |
|  | APC_NM_000038_c.3887delC_p.A1296Efs*9 | 43.20% | 42.53% |  |
| RD_W13_1352_G11 | SMAD4_NM_005359_c.692dupG_p.S232Qfs*3 | 0% | 5.45% | Coverage is 49x for TumorNext run |
|  | TP53_NM_000546_c.97-1G>A_ | 80.35% | 81.56% |  |
| RD_W13_2566_P9 | TP53_NM_000546_c.64delC_p.L22Yfs*22 | 59.77% | 58.62% |  |
| RD_W13_2582_E | BRAF_NM_004333_c.1780G>A_p.D594N | 45.87% | 50.38% |  |
|  | TP53_NM_000546_c.711G>A_p.M237I | 86.12% | 83.13% |  |
| RD_W14_139_D1 | TP53_NM_000546_c.920-2A>C_ | 32.29% | 32.16% |  |
| RD_W14_223_F2 | KRAS_NM_033360_c.35G>A_p.G12D | 27.93% | 27.38% |  |
| RD_W14_531_C1 | TP53_NM_000546_c.743G>A_p.R248Q | 47.48% | 51.07% |  |
| RD_W14_66_A2 | KRAS_NM_033360_c.35G>C_p.G12A | 15.62% | 19.12% |  |
|  | MLH1_NM_000249_c.1489delC_p.R497Gfs*11 | 11.63% | 12.30% |  |
|  | PTEN_NM_000314_c.395G>T_p.G132V | 10.72% | 12.21% |  |
|  | PTEN_NM_000314_c.388C>G_p.R130G | 12.50% | 12.09% |  |
|  | TP53_NM_000546_c.216delC_p.V73Wfs*50 | 10.32% | 8.48% |  |
|  | TP53_NM_000546_c.140C>G_p.P47R | 11.08% | 12.06% |  |
|  | TP53_NM_000546_c.1146delA_p.K382Nfs*40 | 7.07% | 7.59% |  |
|  | TP53_NM_000546_c.214C>G_p.P72A | 10.02% | 8.26% |  |
|  | CCND1_NM_053056_c.842T>A_p.V281E | 4.22% | 5% | Filtered out due to <5% |
| RD_W14_86_B6 | NRAS_NM_002524_c.182A>G_p.Q61R | 33.47% | 36.18% |  |
| RD_W14_958_C1 | PIK3CA_NM_006218_c.3141T>G_p.H1047Q | 22.71% | 31.42% |  |
| RD_W14_298H1 | TP53_NM_000546_c.524G>A_p TP53_NM_000546_c.524G>A_p.R175H | 41% | 40.27% |  |

| **Supplemental Table 3: Results of Accuracy Study for Germline Variant Detection for TumorNext-Lynch-MMR** | | | |
| --- | --- | --- | --- |
| Sample | **gene isoform c_variant p_variant** | Previously Detected Variant Frequency/depth of coverage | Variant Detected by TumorNext-Lynch-MMR Frequency/depth of coverage |
| 16_001 | APC NM_000038 c.5465T>A p.V1822D | 100% 115 | 100.00% 257 |
|  | CDH1 NM_004360 c.184G>A p.G62S | 48.35% 242 | 54.62% 476 |
|  | POLE NM_006231 c.5659G>A p.V1887M | 44.78% 299 | 48.39% 591 |
|  | POLE NM_006231 c.6252A>G p.S2084S | 46.5% 286 | 47.26% 529 |
|  | POLE NM_006231 c.3156G>A p.T1052T | 51.29% 350 | 48.42% 539 |
|  | APC NM_000038 c.1635G>A p.A545A | 100% 102 | 100.00% 292 |
|  | APC NM_000038 c.4479G>A p.T1493T | 100% 234 | 100.00% 386 |
|  | PTEN NM_000314 c.132C>T p.G44G | 57.69% 52 | 52.94% 272 |
|  | MSH6 NM_000179 c.116G>A p.G39E | 100% 370 | 100.00% 582 |
|  | POLE NM_006231 c.4187A>G p.N1396S | 47.3% 297 | 44.31% 580 |
|  | MUTYH NM_001128425 c.64G>A p.V22M | 48.05% 256 | 47.87% 587 |
|  | APC NM_000038 c.5268T>G p.S1756S | 100% 201 | 100.00% 412 |
|  | APC NM_000038 c.5880G>A p.P1960P | 100% 150 | 100.00% 291 |
|  | APC NM_000038 c.1458T>C p.Y486Y | 51.49% 101 | 48.35% 333 |
|  | PMS2 NM_000535 c.780C>G p.S260S | 43.9% 164 | 44.86% 292 |
|  | APC NM_000038 c.5034G>A p.G1678G | 99.55% 223 | 100.00% 311 |
|  | POLE NM_006231 c.4530A>G p.A1510A | 46.25% 326 | 52.20% 546 |
|  |  |  |  |
|  |  |  |  |
| 16_002 | APC NM_000038 c.5465T>A p.V1822D | 100% 113 | 100.00% 241 |
|  | MSH2 NM_000251 c.2732T>G p.L911R | 62.96% 54 | 44.83% 464 |
|  | APC NM_000038 c.1635G>A p.A545A | 100% 83 | 99.76% 423 |
|  | TP53 NM_000546 c.215C>G p.P72R | 99.72% 361 | 100.00% 290 |
|  | APC NM_000038 c.4479G>A p.T1493T | 100% 203 | 100.00% 338 |
|  | MSH6 NM_000179 c.116G>A p.G39E | 46.67% 378 | 47.91% 382 |
|  | APC NM_000038 c.5268T>G p.S1756S | 100% 182 | 100.00% 308 |
|  | MLH1 NM_000249 c.655A>G p.I219V | 47.44% 156 | 48.95% 478 |
|  | APC NM_000038 c.5880G>A p.P1960P | 100% 153 | 99.70% 330 |
|  | APC NM_000038 c.4326T>A p.P1442P | 49.64% 280 | 45.83% 288 |
|  | PMS2 NM_000535 c.780C>G p.S260S | 50.66% 152 | 41.13% 479 |
|  | APC NM_000038 c.1458T>C p.Y486Y | 100% 74 | 100.00% 452 |
|  | MUTYH NM_001128425 c.1187G>A p.G396D | 43.84% 407 | 48.30% 323 |
|  | CDH1 NM_004360 c.2076T>C p.A692A | 99.59% 246 | 100.00% 435 |
|  | APC NM_000038 c.5034G>A p.G1678G | 100% 203 | 100.00% 386 |
|  |  |  |  |
|  |  |  |  |
| 16_003 | APC NM_000038 c.5465T>A p.V1822D | 100% 115 | 100.00% 256 |
|  | MSH6 NM_000179 c.2904C>G p.V968V | 39.16% 167 | 48.71% 349 |
|  | APC NM_000038 c.1635G>A p.A545A | 100% 97 | 100.00% 453 |
|  | TP53 NM_000546 c.215C>G p.P72R | 45.76% 236 | 48.95% 239 |
|  | APC NM_000038 c.4479G>A p.T1493T | 100% 142 | 100.00% 336 |
|  | CDH1 NM_004360 c.88C>A p.P30T | 52.38% 253 | 51.30% 347 |
|  | MSH6 NM_000179 c.540T>C p.D180D | 52.38% 169 | 47.38% 439 |
|  | APC NM_000038 c.5268T>G p.S1756S | 100% 157 | 100.00% 334 |
|  | APC NM_000038 c.5880G>A p.P1960P | 100% 132 | 100.00% 337 |
|  | APC NM_000038 c.4326T>A p.P1442P | 57.07% 208 | 52.08% 288 |
|  | MSH6 NM_000179 c.186C>A p.R62R | 52.16% 233 | 51.93% 337 |
|  | PMS2 NM_000535 c.780C>G p.S260S | 48.03% 127 | 46.68% 482 |
|  | APC NM_000038 c.1458T>C p.Y486Y | 100% 109 | 100.00% 446 |
|  | APC NM_000038 c.5034G>A p.G1678G | 100% 144 | 100.00% 376 |
|  | MSH6 NM_000179 c.276A>G p.P92P | 48.92% 139 | 46.34% 382 |
|  |  |  |  |
|  |  |  |  |
| 16_004 | APC NM_000038 c.5465T>A p.V1822D | 52.05% 171 | 49.87% 389 |
|  | CDH1 NM_004360 c.1896C>T p.H632H | 55.46% 350 | 53.36% 639 |
|  | CDH1 NM_004360 c.2634C>T p.G878G | 49.59% 366 | 53.62% 621 |
|  | POLE NM_006231 c.6252A>G p.S2084S | 100% 290 | 99.84% 627 |
|  | POLE NM_006231 c.3156G>A p.T1052T | 99.72% 357 | 100.00% 619 |
|  | APC NM_000038 c.1635G>A p.A545A | 46.34% 123 | 46.96% 658 |
|  | APC NM_000038 c.4479G>A p.T1493T | 48.78% 288 | 53.30% 469 |
|  | POLE NM_006231 c.4187A>G p.N1396S | 43.93% 323 | 45.83% 611 |
|  | MSH6 NM_000179 c.540T>C p.D180D | 44.96% 282 | 45.64% 642 |
|  | APC NM_000038 c.5268T>G p.S1756S | 48.4% 219 | 46.48% 426 |
|  | APC NM_000038 c.5880G>A p.P1960P | 48.71% 232 | 50.21% 468 |
|  | MSH6 NM_000179 c.186C>A p.R62R | 44.91% 335 | 49.47% 566 |
|  | PMS2 NM_000535 c.780C>G p.S260S | 52.43% 206 | 46.01% 576 |
|  | APC NM_000038 c.1458T>C p.Y486Y | 44.36% 133 | 48.44% 673 |
|  | BMPR1A NM_004329 c.4C>A p.P2T | 45.07% 142 | 48.14% 619 |
|  | CDH1 NM_004360 c.2076T>C p.A692A | 47.86% 352 | 49.92% 645 |
|  | MSH6 NM_000179 c.116G>A p.G39E | 49.87% 373 | 48.10% 578 |
|  | CDH1 NM_004360 c.671G>A p.R224H | 46.06% 255 | 53.20% 594 |
|  | APC NM_000038 c.5034G>A p.G1678G | 44% 275 | 51.26% 517 |
|  | MSH6 NM_000179 c.276A>G p.P92P | 51.21% 248 | 47.30% 649 |
|  | POLE NM_006231 c.4530A>G p.A1510A | 100% 292 | 99.58% 473 |
|  |  |  |  |
|  |  |  |  |
| 16_005 | APC NM_000038 c.5465T>A p.V1822D | 100% 155 | 100.00% 378 |
|  | MUTYH NM_001128425 c.1014G>C p.Q338H | 46.31% 203 | 44.11% 331 |
|  | APC NM_000038 c.1635G>A p.A545A | 100% 76 | 99.50% 398 |
|  | TP53 NM_000546 c.215C>G p.P72R | 100% 214 | 100.00% 376 |
|  | APC NM_000038 c.4479G>A p.T1493T | 100% 197 | 100.00% 368 |
|  | MSH6 NM_000179 c.540T>C p.D180D | 45.13% 196 | 47.83% 460 |
|  | APC NM_000038 c.5268T>G p.S1756S | 100% 177 | 100.00% 354 |
|  | MLH1 NM_000249 c.655A>G p.I219V | 44.67% 150 | 50.57% 437 |
|  | MSH6 NM_000179 c.642C>T p.Y214Y | 43.43% 176 | 48.01% 402 |
|  | APC NM_000038 c.5880G>A p.P1960P | 100% 158 | 100.00% 367 |
|  | PMS2 NM_000535 c.780C>G p.S260S | 52.42% 124 | 50.13% 395 |
|  | APC NM_000038 c.1458T>C p.Y486Y | 100% 121 | 100.00% 387 |
|  | BMPR1A NM_004329 c.4C>A p.P2T | 42.42% 132 | 40.92% 303 |
|  | CDH1 NM_004360 c.2076T>C p.A692A | 47.6% 229 | 46.12% 464 |
|  | APC NM_000038 c.5034G>A p.G1678G | 100% 164 | 100.00% 371 |

| **Supplemental Table 5: Inter-assay reproducibility results for somatic variants** | | | | |
| --- | --- | --- | --- | --- |
| Sample | Variant | Val1 Mutation Freq | Val2 Mutation Freq | Val3 Mutation Freq |
| BR_14_97_07_01_T | APC NM_000038 c.7849G>A p.E2617K | 34.95% | 37.01% | 38.49% |
| BR_14_97_07_01_T | TP53 NM_000546 c.578A>G p.H193R | 54.10% | 56.89% | 58.02% |
| BR-14-131-05-01-T | APC NM_000038 c.3340C>T p.R1114* | 33.16% | 29.37% | 34.23% |
| BR-14-131-05-01-T | APC NM_000038 c.3925G>T p.E1309* | 18.83% | 15.68% | 13.49% |
| BR-14-131-05-01-T | TP53 NM_000546 c.772G>T p.E258* | 44.47% | 34.49% | 50.61% |
| BR-13-162-05-01T | TP53 NM_000546 c.817C>T p.R273C | 14.42% | 15.55% | 15.53% |
| BR-13-162-05-01T | BRAF NM_004333 c.1237G>A p.V413M | 8.74% | 9.93% | 10.55% |
| BR-13-162-05-01T | MSH6 NM_000179 c.3881G>T p.C1294F | 10.79% | 8.68% | 10.75% |
| BR-13-162-05-01T | APC NM_000038 c.4285C>T p.Q1429* | 8.90% | 11.77% | 11.82% |
| BR-13-162-05-01T | APC NM_000038 c.621C>A p.C207* | 11.41% | 9% | 10.93% |
| RD_009 | ERBB2 NM_004448 c.575-3C>T | 53.26% | 55.69% | 56.35% |
| RD_009 | TP53 NM_000546 c.844C>T p.R282W | 51.18% | 49.95% | 45.90% |
| BR-14-283-05-01T | PIK3CA NM_006218 c.337C>G p.L113V | 27.59% | 19.70% | 23.83% |
| BR-14-283-05-01T | APC NM_000038 c.4192_4193delAG p.R1399Ffs*9 | 46.84% | 49.85% | 48.33% |
| BR-14-283-05-01T | PIK3CA NM_006218 c.314_325del12 p.V105_R108del | 20.05% | 14.40% | 17.74% |
| BR-14-283-05-01T | TP53 NM_000546 c.797G>T p.G266V | 56.49% | 47.27% | 53.22% |
| BR-13-184-05-01T | KRAS NM_033360 c.38G>A p.G13D | 77.22% | 72.13% | 75.54% |
| BR-13-184-05-01T | TP53 NM_000546 c.556delG p.D186Mfs*61 | 72.08% | 70.91% | 72.14% |
| BR-13-184-05-01T | APC NM_000038 c.3907C>T p.Q1303* | 66.67% | 71.40% | 71.40% |
| BR-12-110-T | BLM NM_000057 c.1544delA p.N515Mfs*16 | 25.63% | 22.21% | 25.61% |
| BR-12-110-T | POLE NM_006231 c.2469-2A>G | 24.97% | 20.44% | 28.18% |
| BR-12-110-T | POLD1 NM_002691 c.2034_2037del p.D679Pfs*14 | 25.32% | 20.38% | 22.88% |
| BR-12-110-T | AXIN2 NM_004655 c.1474G>A p.A492T | 24.21% | 25.94% | 21.44% |
| BR-12-110-T | PTEN NM_000314 c.800delA p.K267Rfs*9 | 47.41% | 44.63% | 44.52% |
| BR-12-110-T | MSH3 NM_002439 c.857C>A p.P286H | 26.01% | 24.54% | 21.52% |
| BR-12-110-T | MSH3 NM_002439 c.1148delA p.K383Rfs*32 | 26.66% | 24.04% | 23.87% |
| BR-12-110-T | PALB2 NM_024675 c.1471G>A p.A491T | 22.01% | 23.74% | 20.85% |
| BR-12-110-T | MSH3 NM_002439 c.2216dupA p.N739Kfs*29 | 20.05% | 15.79% | 19.22% |
| BR-12-110-T | BRAF NM_004333 c.1208delC p.P403Lfs*8 | 27.16% | 22.03% | 23.11% |
| BR-12-110-T | KRAS NM_033360 c.35G>A p.G12D | 46.72% | 48.16% | 45.93% |
| BR-12-110-T | APC NM_000038 c.423-5_423-4delAA | 33.41% | 21.68% | 24.14% |
| BR-14-239-05-01-T | TP53 NM_000546 c.713G>T p.C238F | 39.05% | 32.07% | 33.75% |
| BR-14-239-05-01-T | PIK3CA NM_006218 c.1633G>A p.E545K | 12.39% | 8.73% | 8.17% |
| BR-14-239-05-01-T | APC NM_000038 c.1935_1951del17 p.I646Pfs*22 | 16.01% | 16.57% | 15.77% |
| BR-14-239-05-01-T | KRAS NM_033360 c.35G>T p.G12V | 49.58% | 48.21% | 49.71% |
| BR-11-93-T | TP53 NM_000546 c.404G>T p.C135F | 26.80% | 28.96% | 30.08% |

| **Supplemental Table 6: Inter-assay reproducibility results for germline variants** | | | | |
| --- | --- | --- | --- | --- |
| Sample | Variant | Val1 Frequency/depth of coverage | Val2 Frequency/depth of coverage | Val3 Frequency/depth of coverage |
| BR_14_97_07_01_T | APC NM_000038 c.5465T>A p.V1822D | 100% 145 | 99.49% 395 | 99.62% 260 |
| BR_14_97_07_01_T | POLE NM_006231 c.6252A>G p.S2084S | 100% 290 | 99.86% 712 | 100% 273 |
| BR_14_97_07_01_T | POLE NM_006231 c.3156G>A p.T1052T | 100% 331 | 100% 1000 | 100% 397 |
| BR_14_97_07_01_T | APC NM_000038 c.1635G>A p.A545A | 100% 94 | 100% 220 | 100% 181 |
| BR_14_97_07_01_T | TP53 NM_000546 c.215C>G p.P72R | 100% 274 | 100% 1004 | 100% 346 |
| BR_14_97_07_01_T | APC NM_000038 c.4479G>A p.T1493T | 100% 260 | 99.88% 848 | 100% 386 |
| BR_14_97_07_01_T | PMS2 NM_000535 c.1621A>G p.E541E | 38.98% 237 | 46.6% 1151 | 45.19% 375 |
| BR_14_97_07_01_T | MSH6 NM_000179 c.116G>A p.G39E | 46.12% 259 | 47.3% 1151 | 48.94% 378 |
| BR_14_97_07_01_T | POLE NM_006231 c.4187A>G p.N1396S | 48.4% 281 | 46.76% 788 | 49.07% 270 |
| BR_14_97_07_01_T | APC NM_000038 c.5268T>G p.S1756S | 99.48% 194 | 99.89% 892 | 99.68% 316 |
| BR_14_97_07_01_T | POLD1 NM_002691 c.2244T>C p.S748S | 43.34% 293 | 46.28% 810 | 49.41% 339 |
| BR_14_97_07_01_T | APC NM_000038 c.5880G>A p.P1960P | 100% 217 | 99.84% 617 | 100% 353 |
| BR_14_97_07_01_T | MSH6 NM_000179 c.3727A>T p.T1243S | 38.76% 209 | 46.94% 443 | 44.82% 328 |
| BR_14_97_07_01_T | APC NM_000038 c.1458T>C p.Y486Y | 100% 197 | 100% 288 | 99.58% 237 |
| BR_14_97_07_01_T | PMS2 NM_000535 c.1408C>T p.P470S | 42.46% 253 | 46.86% 1259 | 47.74% 356 |
| BR_14_97_07_01_T | CDH1 NM_004360 c.2076T>C p.A692A | 100% 238 | 100% 909 | 100% 346 |
| BR_14_97_07_01_T | POLD1 NM_002691 c.810T>C p.A270A | 52.09% 263 | 44.71% 1165 | 46.33% 354 |
| BR_14_97_07_01_T | APC NM_000038 c.5034G>A p.G1678G | 100% 270 | 99.89% 900 | 100% 326 |
| BR_14_97_07_01_T | POLE NM_006231 c.4530A>G p.A1510A | 100% 252 | 99.87% 773 | 100% 280 |
| BR-14-131-05-01-T | APC NM_000038 c.5465T>A p.V1822D | 100% 251 | 99.88% 868 | 100% 173 |
| BR-14-131-05-01-T | MUTYH NM_001128425 c.1014G>C p.Q338H | 47.01% 520 | 47.29% 832 | 46.1% 564 |
| BR-14-131-05-01-T | APC NM_000038 c.1635G>A p.A545A | 100% 189 | 99.79% 469 | 100% 111 |
| BR-14-131-05-01-T | TP53 NM_000546 c.215C>G p.P72R | 45.79% 537 | 45.9% 1335 | 48.28% 671 |
| BR-14-131-05-01-T | APC NM_000038 c.4479G>A p.T1493T | 100% 463 | 100% 1187 | 99.57% 235 |
| BR-14-131-05-01-T | PMS2 NM_000535 c.1621A>G p.E541E | 100% 413 | 100% 1215 | 99.77% 435 |
| BR-14-131-05-01-T | PMS2 NM_000535 c.2570G>C p.G857A | 68.49% 73 | 68.52% 54 | 72.73% 33 |
| BR-14-131-05-01-T | MSH2 NM_000251 c.2006-6T>C | 52.07% 364 | 47.24% 708 | 40% 136 |
| BR-14-131-05-01-T | PMS2 NM_000535 c.288C>T p.A96A | 49.19% 246 | 42.33% 808 | 48.86% 88 |
| BR-14-131-05-01-T | PMS2 NM_000535 c.1454C>A p.T485K | 45.37% 518 | 52.28% 1381 | 46.56% 568 |
| BR-14-131-05-01-T | APC NM_000038 c.5268T>G p.S1756S | 99.73% 377 | 99.81% 1079 | 100% 162 |
| BR-14-131-05-01-T | APC NM_000038 c.5880G>A p.P1960P | 100% 381 | 100% 1059 | 100% 137 |
| BR-14-131-05-01-T | PMS2 NM_000535 c.379G>A p.A127T | 45.22% 431 | 47.19% 679 | 45% 222 |
| BR-14-131-05-01-T | PMS2 NM_000535 c.780C>G p.S260S | 100% 186 | 100% 644 | 100% 168 |
| BR-14-131-05-01-T | APC NM_000038 c.1458T>C p.Y486Y | 100% 309 | 100% 593 | 100% 84 |
| BR-14-131-05-01-T | BMPR1A NM_004329 c.4C>A p.P2T | 42.12% 312 | 43.03% 586 | 48.11% 107 |
| BR-14-131-05-01-T | PMS2 NM_000535 c.2007-4G>A | 39.02% 124 | 39.22% 232 | 44.12% 138 |
| BR-14-131-05-01-T | PMS2 NM_000535 c.1408C>T p.P470S | 48.48% 528 | 45.19% 1433 | 50.42% 482 |
| BR-14-131-05-01-T | APC NM_000038 c.5034G>A p.G1678G | 100% 476 | 100% 1148 | 100% 191 |
| BR-13-162-05-01T | APC NM_000038 c.5465T>A p.V1822D | 46.9% 291 | 44.81% 272 | 46.94% 196 |
| BR-13-162-05-01T | MUTYH NM_001128425 c.1014G>C p.Q338H | 50% 152 | 52% 125 | 53.09% 81 |
| BR-13-162-05-01T | POLE NM_006231 c.6252A>G p.S2084S | 42.08% 183 | 50.26% 194 | 47.01% 118 |
| BR-13-162-05-01T | POLE NM_006231 c.3156G>A p.T1052T | 43.95% 249 | 43.55% 287 | 57.34% 143 |
| BR-13-162-05-01T | APC NM_000038 c.1635G>A p.A545A | 47.06% 153 | 41.29% 155 | 51.16% 86 |
| BR-13-162-05-01T | TP53 NM_000546 c.215C>G p.P72R | 100% 221 | 100% 241 | 100% 135 |
| BR-13-162-05-01T | APC NM_000038 c.4479G>A p.T1493T | 50.81% 246 | 48.35% 246 | 47.1% 155 |
| BR-13-162-05-01T | PMS2 NM_000535 c.1621A>G p.E541E | 39.29% 196 | 39% 302 | 43.28% 134 |
| BR-13-162-05-01T | APC NM_000038 c.2322C>T p.D774D | 40.53% 228 | 47.44% 215 | 46.27% 134 |
| BR-13-162-05-01T | MSH6 NM_000179 c.540T>C p.D180D | 44.39% 197 | 48.28% 145 | 47.96% 98 |
| BR-13-162-05-01T | APC NM_000038 c.5268T>G p.S1756S | 54.22% 225 | 45.23% 243 | 54.26% 129 |
| BR-13-162-05-01T | APC NM_000038 c.5880G>A p.P1960P | 40.14% 279 | 44.04% 277 | 45.61% 171 |
| BR-13-162-05-01T | MSH6 NM_000179 c.186C>A p.R62R | 52.41% 166 | 42.86% 238 | 53.12% 97 |
| BR-13-162-05-01T | POLE NM_006231 c.6418G>A p.E2140K | 44.88% 205 | 48.02% 228 | 45.16% 124 |
| BR-13-162-05-01T | PMS2 NM_000535 c.780C>G p.S260S | 52.71% 130 | 39.04% 146 | 46.05% 76 |
| BR-13-162-05-01T | APC NM_000038 c.1458T>C p.Y486Y | 49.09% 220 | 56.14% 173 | 48.18% 110 |
| BR-13-162-05-01T | CDH1 NM_004360 c.2076T>C p.A692A | 54.8% 177 | 50.56% 178 | 51.46% 103 |
| BR-13-162-05-01T | PMS2 NM_000535 c.1408C>T p.P470S | 46.83% 206 | 49.46% 278 | 45.45% 155 |
| BR-13-162-05-01T | APC NM_000038 c.5034G>A p.G1678G | 47.3% 241 | 42.63% 251 | 40.15% 137 |
| BR-13-162-05-01T | MSH6 NM_000179 c.276A>G p.P92P | 34.68% 173 | 51.49% 135 | 44.04% 109 |
| BR-13-162-05-01T | POLE NM_006231 c.4530A>G p.A1510A | 55.63% 152 | 47.25% 182 | 47.62% 84 |
| RD_009 | APC NM_000038 c.5465T>A p.V1822D | 40.97% 395 | 47.34% 606 | 49.82% 543 |
| RD_009 | APC NM_000038 c.1635G>A p.A545A | 48.15% 352 | 46.75% 338 | 43.93% 305 |
| RD_009 | TP53 NM_000546 c.215C>G p.P72R | 100% 483 | 100% 599 | 100% 451 |
| RD_009 | APC NM_000038 c.4479G>A p.T1493T | 49.11% 507 | 42.41% 582 | 47.32% 561 |
| RD_009 | PMS2 NM_000535 c.1621A>G p.E541E | 100% 428 | 100% 578 | 100% 462 |
| RD_009 | PMS2 NM_000535 c.2570G>C p.G857A | 51.81% 83 | 57.14% 21 | 32.65% 49 |
| RD_009 | MSH6 NM_000179 c.540T>C p.D180D | 49.79% 471 | 47.67% 453 | 45.27% 402 |
| RD_009 | APC NM_000038 c.5268T>G p.S1756S | 42.24% 490 | 44.14% 666 | 46.98% 562 |
| RD_009 | PMS2 NM_000535 c.2466T>C p.L822L | 45.45% 33 | 48.44% 64 | 45% 60 |
| RD_009 | APC NM_000038 c.5880G>A p.P1960P | 46.85% 508 | 48.95% 620 | 44.68% 527 |
| RD_009 | MSH6 NM_000179 c.186C>A p.R62R | 52.17% 512 | 45.61% 692 | 50.57% 441 |
| RD_009 | POLE NM_006231 c.6418G>A p.E2140K | 50.18% 548 | 48.07% 699 | 51.67% 539 |
| RD_009 | CDH1 NM_004360 c.2253C>T p.N751N | 50.09% 589 | 48.76% 607 | 48.83% 513 |
| RD_009 | PMS2 NM_000535 c.780C>G p.S260S | 99.55% 223 | 100% 289 | 100% 331 |
| RD_009 | APC NM_000038 c.1458T>C p.Y486Y | 48.23% 455 | 42.13% 396 | 50.68% 365 |
| RD_009 | BMPR1A NM_004329 c.4C>A p.P2T | 42.12% 523 | 42.89% 460 | 41.73% 418 |
| RD_009 | PMS2 NM_000535 c.2007-4G>A | 29.63% 136 | 50.54% 93 | 41.55% 143 |
| RD_009 | CDH1 NM_004360 c.2076T>C p.A692A | 100% 444 | 99.79% 468 | 100% 392 |
| RD_009 | PMS2 NM_000535 c.1408C>T p.P470S | 100% 450 | 100% 700 | 100% 444 |
| RD_009 | APC NM_000038 c.5034G>A p.G1678G | 45.02% 542 | 45.53% 584 | 48.18% 496 |
| RD_009 | MSH6 NM_000179 c.276A>G p.P92P | 47.56% 532 | 50.39% 509 | 50.45% 442 |
| BR-14-283-05-01T | APC NM_000038 c.5465T>A p.V1822D | 100% 106 | 100% 182 | 100% 202 |
| BR-14-283-05-01T | POLE NM_006231 c.6252A>G p.S2084S | 100% 67 | 100% 104 | 100% 72 |
| BR-14-283-05-01T | POLE NM_006231 c.3156G>A p.T1052T | 100% 50 | 100% 117 | 100% 99 |
| BR-14-283-05-01T | APC NM_000038 c.1635G>A p.A545A | 100% 52 | 100% 73 | 100% 71 |
| BR-14-283-05-01T | TP53 NM_000546 c.215C>G p.P72R | 50% 50 | 50% 114 | 42.72% 103 |
| BR-14-283-05-01T | APC NM_000038 c.4479G>A p.T1493T | 100% 94 | 100% 169 | 100% 187 |
| BR-14-283-05-01T | PMS2 NM_000535 c.1621A>G p.E541E | 59.18% 49 | 44.25% 113 | 48.15% 108 |
| BR-14-283-05-01T | APC NM_000038 c.5268T>G p.S1756S | 100% 79 | 100% 165 | 100% 149 |
| BR-14-283-05-01T | MLH1 NM_000249 c.655A>G p.I219V | 52.04% 102 | 32.73% 112 | 45.45% 143 |
| BR-14-283-05-01T | APC NM_000038 c.5880G>A p.P1960P | 100% 73 | 100% 172 | 100% 178 |
| BR-14-283-05-01T | CDH1 NM_004360 c.2253C>T p.N751N | 47.37% 76 | 42.94% 165 | 48.08% 105 |
| BR-14-283-05-01T | PMS2 NM_000535 c.780C>G p.S260S | 48.84% 43 | 35.45% 110 | 51.11% 90 |
| BR-14-283-05-01T | APC NM_000038 c.1458T>C p.Y486Y | 39.8% 98 | 43.37% 168 | 45.39% 143 |
| BR-14-283-05-01T | CDH1 NM_004360 c.2076T>C p.A692A | 50.98% 51 | 54.33% 127 | 43.43% 99 |
| BR-14-283-05-01T | PMS2 NM_000535 c.1408C>T p.P470S | 47.62% 63 | 40.91% 132 | 40.91% 88 |
| BR-14-283-05-01T | APC NM_000038 c.5034G>A p.G1678G | 99.03% 104 | 100% 155 | 100% 138 |
| BR-14-283-05-01T | POLE NM_006231 c.4530A>G p.A1510A | 100% 28 | 98.96% 96 | 100% 108 |
| BR-13-184-05-01T | POLD1 NM_002691 c.1485C>T p.T495T | 48.44% 995 | 46.9% 550 | 48.92% 186 |
| BR-13-184-05-01T | POLE NM_006231 c.6252A>G p.S2084S | 46.82% 866 | 44.18% 507 | 45.57% 158 |
| BR-13-184-05-01T | POLE NM_006231 c.3156G>A p.T1052T | 49% 1004 | 50.63% 633 | 50.45% 220 |
| BR-13-184-05-01T | PMS2 NM_000535 c.1621A>G p.E541E | 100% 726 | 99.68% 633 | 100% 179 |
| BR-13-184-05-01T | PMS2 NM_000535 c.1569C>G p.S523S | 43.45% 887 | 46.86% 735 | 55.98% 209 |
| BR-13-184-05-01T | MLH1 NM_000249 c.655A>G p.I219V | 48.35% 668 | 48.11% 503 | 40% 175 |
| BR-13-184-05-01T | PMS2 NM_000535 c.780C>G p.S260S | 100% 372 | 99.43% 353 | 100% 133 |
| BR-13-184-05-01T | CDH1 NM_004360 c.2076T>C p.A692A | 46.95% 869 | 46.89% 563 | 45.5% 200 |
| BR-13-184-05-01T | PMS2 NM_000535 c.1408C>T p.P470S | 100% 909 | 100% 712 | 100% 190 |
| BR-13-184-05-01T | POLE NM_006231 c.4530A>G p.A1510A | 48.86% 967 | 48.26% 575 | 50.49% 206 |
| BR-13-116-05-01T | APC NM_000038 c.5465T>A p.V1822D | 46.23% 292 | 46.82% 1104 | 47.56% 781 |
| BR-13-116-05-01T | MUTYH NM_001128425 c.1014G>C p.Q338H | 100% 296 | 100% 509 | 100% 532 |
| BR-13-116-05-01T | POLD1 NM_002691 c.356G>A p.R119H | 48.18% 358 | 50.81% 558 | 51.76% 427 |
| BR-13-116-05-01T | POLE NM_006231 c.6252A>G p.S2084S | 99.71% 348 | 100% 578 | 100% 529 |
| BR-13-116-05-01T | POLE NM_006231 c.3156G>A p.T1052T | 100% 377 | 100% 806 | 100% 657 |
| BR-13-116-05-01T | APC NM_000038 c.1635G>A p.A545A | 46.8% 203 | 45.62% 653 | 44.6% 491 |
| BR-13-116-05-01T | TP53 NM_000546 c.215C>G p.P72R | 43.66% 340 | 47.45% 908 | 47.65% 683 |
| BR-13-116-05-01T | APC NM_000038 c.4479G>A p.T1493T | 45.56% 360 | 46.95% 1118 | 48.52% 714 |
| BR-13-116-05-01T | PMS2 NM_000535 c.1621A>G p.E541E | 100% 280 | 100% 862 | 100% 565 |
| BR-13-116-05-01T | PMS2 NM_000535 c.2570G>C p.G857A | 100% 38 | 100% 24 | 100% 51 |
| BR-13-116-05-01T | MSH2 NM_000251 c.2006-6T>C | 44.19% 353 | 47.05% 661 | 45.01% 535 |
| BR-13-116-05-01T | MSH6 NM_000179 c.540T>C p.D180D | 49.35% 384 | 47.06% 904 | 41.07% 600 |
| BR-13-116-05-01T | APC NM_000038 c.5268T>G p.S1756S | 44.38% 338 | 44.92% 1073 | 49.79% 729 |
| BR-13-116-05-01T | PMS2 NM_000535 c.2466T>C p.L822L | 34.62% 26 | 40.16% 122 | 53.62% 69 |
| BR-13-116-05-01T | MLH1 NM_000249 c.655A>G p.I219V | 46.44% 296 | 47.08% 824 | 48.15% 705 |
| BR-13-116-05-01T | APC NM_000038 c.5880G>A p.P1960P | 45.89% 377 | 46.57% 1225 | 48.52% 778 |
| BR-13-116-05-01T | POLD1 NM_002691 c.324G>A p.A108A | 49.27% 342 | 56.54% 453 | 52.07% 387 |
| BR-13-116-05-01T | MSH6 NM_000179 c.186C>A p.R62R | 46.63% 342 | 48.45% 585 | 49.45% 553 |
| BR-13-116-05-01T | PMS2 NM_000535 c.780C>G p.S260S | 99.35% 155 | 99.82% 544 | 100% 437 |
| BR-13-116-05-01T | APC NM_000038 c.1458T>C p.Y486Y | 40.92% 325 | 46.37% 676 | 46.6% 515 |
| BR-13-116-05-01T | BMPR1A NM_004329 c.4C>A p.P2T | 42.94% 338 | 46.09% 846 | 46.79% 640 |
| BR-13-116-05-01T | PMS2 NM_000535 c.2007-4G>A | 98.9% 91 | 85.29% 171 | 90.5% 200 |
| BR-13-116-05-01T | CDH1 NM_004360 c.2076T>C p.A692A | 42.56% 338 | 46.09% 897 | 47.42% 602 |
| BR-13-116-05-01T | PMS2 NM_000535 c.1408C>T p.P470S | 100% 330 | 99.9% 970 | 100% 648 |
| BR-13-116-05-01T | APC NM_000038 c.5034G>A p.G1678G | 48.1% 447 | 44.12% 1024 | 46.37% 757 |
| BR-13-116-05-01T | MSH6 NM_000179 c.276A>G p.P92P | 55.25% 363 | 44.36% 800 | 46.32% 571 |
| BR-13-116-05-01T | POLE NM_006231 c.4530A>G p.A1510A | 100% 331 | 99.86% 715 | 100% 505 |
| BR-12-110-T | APC NM_000038 c.5465T>A p.V1822D | 100% 172 | 100% 347 | 100% 269 |
| BR-12-110-T | POLD1 NM_002691 c.2678A>G p.D893G | 38.64% 265 | 43.91% 477 | 41.13% 265 |
| BR-12-110-T | MUTYH NM_001128425 c.1014G>C p.Q338H | 44.25% 226 | 42.44% 272 | 47.64% 212 |
| BR-12-110-T | POLE NM_006231 c.6252A>G p.S2084S | 100% 229 | 99.38% 324 | 100% 218 |
| BR-12-110-T | POLE NM_006231 c.3156G>A p.T1052T | 100% 242 | 100% 433 | 100% 313 |
| BR-12-110-T | APC NM_000038 c.1635G>A p.A545A | 100% 174 | 100% 187 | 100% 157 |
| BR-12-110-T | TP53 NM_000546 c.215C>G p.P72R | 43.33% 272 | 46.25% 413 | 51.95% 257 |
| BR-12-110-T | APC NM_000038 c.4479G>A p.T1493T | 100% 249 | 100% 433 | 100% 303 |
| BR-12-110-T | PMS2 NM_000535 c.1621A>G p.E541E | 100% 185 | 100% 422 | 100% 252 |
| BR-12-110-T | MSH6 NM_000179 c.116G>A p.G39E | 48.93% 233 | 44.27% 497 | 51.72% 261 |
| BR-12-110-T | MSH2 NM_000251 c.2006-6T>C | 45.07% 213 | 43.85% 244 | 43.78% 234 |
| BR-12-110-T | PMS2 NM_000535 c.288C>T p.A96A | 100% 206 | 100% 306 | 99.44% 180 |
| BR-12-110-T | PMS2 NM_000535 c.1454C>A p.T485K | 100% 204 | 100% 465 | 100% 226 |
| BR-12-110-T | APC NM_000038 c.5268T>G p.S1756S | 100% 217 | 100% 392 | 100% 240 |
| BR-12-110-T | APC NM_000038 c.5880G>A p.P1960P | 100% 233 | 100% 402 | 100% 284 |
| BR-12-110-T | MSH6 NM_000179 c.3306T>A p.T1102T | 44.93% 279 | 48.24% 398 | 48.25% 259 |
| BR-12-110-T | PMS2 NM_000535 c.780C>G p.S260S | 100% 125 | 99.63% 270 | 100% 157 |
| BR-12-110-T | APC NM_000038 c.1458T>C p.Y486Y | 48.42% 223 | 52.31% 325 | 49.5% 200 |
| BR-12-110-T | BMPR1A NM_004329 c.4C>A p.P2T | 99.54% 218 | 100% 266 | 100% 258 |
| BR-12-110-T | CDH1 NM_004360 c.2076T>C p.A692A | 51.93% 233 | 47.58% 414 | 41.42% 240 |
| BR-12-110-T | APC NM_000038 c.5034G>A p.G1678G | 100% 238 | 100% 383 | 100% 253 |
| BR-12-110-T | POLE NM_006231 c.4530A>G p.A1510A | 100% 242 | 100% 405 | 100% 235 |
| BR-14-239-05-01-T | APC NM_000038 c.5465T>A p.V1822D | 100% 530 | 100% 717 | 100% 665 |
| BR-14-239-05-01-T | MUTYH NM_001128425 c.1014G>C p.Q338H | 97.3% 593 | 99.77% 443 | 99.8% 503 |
| BR-14-239-05-01-T | POLE NM_006231 c.6252A>G p.S2084S | 45.1% 776 | 44.26% 549 | 45.42% 496 |
| BR-14-239-05-01-T | POLE NM_006231 c.3156G>A p.T1052T | 47.53% 728 | 49.6% 754 | 50.33% 601 |
| BR-14-239-05-01-T | APC NM_000038 c.1635G>A p.A545A | 100% 398 | 99.78% 455 | 100% 318 |
| BR-14-239-05-01-T | TP53 NM_000546 c.215C>G p.P72R | 45.66% 614 | 42.53% 826 | 49.11% 622 |
| BR-14-239-05-01-T | APC NM_000038 c.4479G>A p.T1493T | 100% 717 | 99.87% 782 | 100% 595 |
| BR-14-239-05-01-T | POLE NM_006231 c.91G>T p.A31S | 44.31% 601 | 46.41% 656 | 48.23% 479 |
| BR-14-239-05-01-T | PMS2 NM_000535 c.1621A>G p.E541E | 43.61% 629 | 46.13% 904 | 49.2% 626 |
| BR-14-239-05-01-T | MSH6 NM_000179 c.540T>C p.D180D | 44.67% 699 | 46.02% 628 | 49.21% 504 |
| BR-14-239-05-01-T | APC NM_000038 c.5268T>G p.S1756S | 100% 520 | 99.87% 769 | 100% 650 |
| BR-14-239-05-01-T | APC NM_000038 c.5880G>A p.P1960P | 99.85% 657 | 100% 849 | 100% 718 |
| BR-14-239-05-01-T | MSH6 NM_000179 c.186C>A p.R62R | 48.05% 516 | 46.74% 645 | 46.09% 628 |
| BR-14-239-05-01-T | CDH1 NM_004360 c.2253C>T p.N751N | 45.6% 811 | 46.3% 787 | 45.27% 584 |
| BR-14-239-05-01-T | PMS2 NM_000535 c.780C>G p.S260S | 50% 328 | 44.59% 531 | 47.88% 425 |
| BR-14-239-05-01-T | APC NM_000038 c.1458T>C p.Y486Y | 100% 633 | 100% 468 | 100% 436 |
| BR-14-239-05-01-T | BMPR1A NM_004329 c.4C>A p.P2T | 45.25% 732 | 41.21% 600 | 47.46% 555 |
| BR-14-239-05-01-T | CDH1 NM_004360 c.2076T>C p.A692A | 93.39% 591 | 100% 646 | 100% 531 |
| BR-14-239-05-01-T | PMS2 NM_000535 c.1408C>T p.P470S | 52.54% 690 | 43.52% 914 | 47.34% 660 |
| BR-14-239-05-01-T | APC NM_000038 c.5034G>A p.G1678G | 100% 691 | 100% 747 | 99.86% 690 |
| BR-14-239-05-01-T | MSH6 NM_000179 c.276A>G p.P92P | 49.86% 708 | 45.02% 603 | 45.83% 515 |
| BR-14-239-05-01-T | POLE NM_006231 c.4530A>G p.A1510A | 38.83% 633 | 43.14% 653 | 46.32% 517 |
| BR-11-93-T | APC NM_000038 c.5465T>A p.V1822D | 100% 208 | 100% 278 | 100% 243 |
| BR-11-93-T | MUTYH NM_001128425 c.1014G>C p.Q338H | 100% 288 | 100% 265 | 100% 182 |
| BR-11-93-T | POLE NM_006231 c.6252A>G p.S2084S | 100% 317 | 99.56% 225 | 100% 193 |
| BR-11-93-T | POLE NM_006231 c.3156G>A p.T1052T | 100% 368 | 99.7% 334 | 100% 258 |
| BR-11-93-T | APC NM_000038 c.1635G>A p.A545A | 100% 168 | 100% 197 | 100% 131 |
| BR-11-93-T | TP53 NM_000546 c.215C>G p.P72R | 46.76% 293 | 45.93% 383 | 37.73% 274 |
| BR-11-93-T | APC NM_000038 c.4479G>A p.T1493T | 100% 316 | 100% 370 | 100% 240 |
| BR-11-93-T | PMS2 NM_000535 c.1621A>G p.E541E | 100% 303 | 99.7% 329 | 100% 268 |
| BR-11-93-T | PMS2 NM_000535 c.288C>T p.A96A | 99.61% 260 | 100% 219 | 100% 175 |
| BR-11-93-T | PMS2 NM_000535 c.1454C>A p.T485K | 100% 347 | 100% 404 | 100% 260 |
| BR-11-93-T | APC NM_000038 c.5268T>G p.S1756S | 99.6% 248 | 100% 297 | 100% 261 |
| BR-11-93-T | POLD1 NM_002691 c.2527C>T p.L843L | 46.94% 294 | 48.02% 253 | 53.73% 202 |
| BR-11-93-T | APC NM_000038 c.5880G>A p.P1960P | 100% 287 | 99.72% 363 | 100% 245 |
| BR-11-93-T | PMS2 NM_000535 c.780C>G p.S260S | 100% 153 | 100% 187 | 100% 164 |
| BR-11-93-T | APC NM_000038 c.1458T>C p.Y486Y | 100% 233 | 100% 207 | 100% 200 |
| BR-11-93-T | BMPR1A NM_004329 c.4C>A p.P2T | 45.61% 297 | 41.5% 255 | 42.79% 202 |
| BR-11-93-T | CDH1 NM_004360 c.2076T>C p.A692A | 45.04% 262 | 42.9% 310 | 44.96% 258 |
| BR-11-93-T | APC NM_000038 c.5034G>A p.G1678G | 100% 368 | 99.69% 325 | 100% 242 |
| BR-11-93-T | POLE NM_006231 c.4530A>G p.A1510A | 100% 312 | 100% 297 | 100% 205 |
